# A GC-MS Chemotaxonomic Study on Lipophilic Compounds in the Bark of *S. aucuparia* subsp. *sibirica* Trees from the Population Growing in Akademgorodok, Novosibirsk (Russia)

**DOI:** 10.3390/metabo13060768

**Published:** 2023-06-19

**Authors:** Asya R. Vasilieva, Nikolay M. Slynko, Ljudmila E. Tatarova, Vadim M. Efimov, Leonid V. Kuibida, Sergey V. Asbaganov, Sergey E. Peltek

**Affiliations:** 1 Federal Research Center, Institute of Cytology and Genetics of SB RAS, 630090 Novosibirsk, Russia; nslynko@mail.ru (N.M.S.); ljudatat@mail.ru (L.E.T.); efimov@bionet.nsc.ru (V.M.E.); peltek@bionet.nsc.ru (S.E.P.); 2 Kurchatov Genomic Center, Federal Research Center ICG, SB RAS, 630090 Novosibirsk, Russia; 3 Institute of Chemical Kinetics and Combustion of SB RAS, 630090 Novosibirsk, Russia; kuibida@kinetics.nsc.ru; 4 Central Siberian Botanical Garden of SB RAS, 630090 Novosibirsk, Russia; cryonus@mail.ru

**Keywords:** sorbus bark, metabolites, chemotaxonomy, population, GS-MS

## Abstract

Determination of chemotypes and of their role in the polymorphism of populations is an important field in the research on secondary metabolites of plants. In the present study, by gas chromatography coupled with mass spectrometry, the composition of bark extracts from rowan *S. aucuparia* subsp. *sibirica* was determined for 16 trees growing within Akademgorodok of Novosibirsk, with bark samples collected both in winter and summer. Among 101 fully or partially identified metabolites, there are alkanes, alkenes, linear alcohols, fatty acids and their derivatives, phenols and their derivatives, prunasin and its parent and derivative compounds, polyprenes and their derivatives, cyclic diterpenes, and phytosterols. These compounds were grouped according to their biosynthesis pathways. Cluster analysis revealed two groups among the bark samples collected in winter and three groups among bark samples collected in summer. The key determinants of this clustering are the biosynthesis of metabolites via the cyanogenic pathway (especially potentially toxic prunasin) and their formation via the phytosterol pathway (especially potentially pharmacologically useful lupeol). It follows from the results that the presence of chemotypes having sharply different profiles of metabolites in a population from a small geographic area invalidates the practice of general sampling to obtain averaged data when a population is described. From the standpoint of possible industrial use or plant selection based on metabolomic data, it is possible to select specific sets of samples containing a minimal amount of potentially toxic compounds and the largest amount of potentially useful substances.

## 1. Introduction

A systematic study on low-molecular-weight metabolites identified in a biological sample helps to understand the so-called metabolome. In particular, metabolomics allows to describe the molecular phenotype of organisms most accurately and comprehensively. Such a description may be useful for chemotaxonomic classification of specific organisms by means of similarities and differences in their metabolic profiles.

The use of chemotaxonomy can considerably supplement—and often alter—conclusions based on the traditional binomial classification system. Chemotaxonomic differentiation between living creatures can be achieved if there is a sufficient amount of experimental data confirming reproducibility of the chemical composition of the studied species.

The use of chemotaxonomy to classify plants allows researchers to introduce one more level of chemico-biological organization. Chemotypes are a set of morphologically similar plants having different profiles of secondary metabolites. This composition changes during stages of plant development, either owing to variation in growing conditions or as a result of structural or physiological modifications in the plant under the influence of specific environmental factors (phenotypic plasticity) [1,2]. Thus, differentiation between chemotypes requires a detailed analysis of secondary metabolites as well as molecular biological assays and statistical methods.

One of the most intricate and taxonomically complex genera is *Sorbus* L. of the tribe Maleae Small., which includes ~250 species [3]. Interaction of effects of interspecific and intergeneric hybridization, polyploidy, and apomixis as well as a wide variation of morphological characteristics greatly complicate the investigation of phyletic relationships and taxonomy of this genus [4,5,6].

*Sorbus aucuparia* L. s.l. is the most widespread and polymorphic species of the genus and is common in northern regions and been of the most cold-resistant woody plants. According to chorological data [7], the trans-Eurasian geographical range of *S. aucuparia* covers most of the temperate zone of Eurasia and of nearby islands, rising to the upper boundary of the forest belt in the mountains. On such a large geographical range, *S. aucuparia* s.l. has been differentiated into a number of subspecific taxa. For Siberia, it is customary to highlight *S. aucuparia* subsp. *sibirica* (Hedl.) Krylov, which is distinguished by weakly pubescent or almost bare leaves and petioles, weakly expressed keel and pockets on leaf veins, and sparsely pubescent buds [8,9]. However, depending on the latitude of the habitat and environmental conditions, Siberian rowan populations may more or less deviate towards *S. aucuparia* s.str. or can combine features of several subtaxa [10]. Populations from the vicinity of Akademgorodok are characterized by the manifestation of intermediate traits [9].

Rowan is often used as an ornamental and medicinal plant, and less often as a food plant [11,12]. Our comparative analysis included the Nevezhinskaya food variety zoned in Novosibirsk Oblast, a sweet-fruited natural genotype of *S. aucuparia* s.str. from Vladimir Oblast in the Russian Federation [13] and is regularly seen in cultivated plantings in Novosibirsk Oblast.

Lately, in the era of active climatic fluctuations and increasing economic activity in northern regions, underutilized plants are increasingly receiving attention as sources of components of functional foods, nutraceuticals, and cosmeceuticals.

The aim of this study was to create a chemotaxonomic classification of the population of Siberian rowan, *S. aucuparia* subsp. *sibirica*, growing in the upper zone of Akademgorodok in Novosibirsk by comparative profiling of lipophilic metabolites in bark extracts, taking into account the influence of seasonal changes.

## 2. Materials and Methods

### 2.1. Plant Materials

Summer sampling was carried out in May–July 2021; winter samples were collected in January–February 2022. In addition, to examine the seasonal changes, samples were selectively taken in the off-season period: April 2022.

Twigs with a diameter of 0.6–0.8 cm were cut off from distal parts of lower branches of an individual tree, and the peridermal layer was removed from the branches with a knife.

The geographical location of the collected samples is given in the Supplementary Materials (Table S1).

### 2.2. Reagents and Solvents

All chemical reagents and solvents (hypergrade for LS-MS) used in this study were obtained from Sigma-Aldrich, Inc. (Steinheim, Germany). Lupeol, luphenone, betulin, and betulin aldehyde, used as standards, were kindly provided by the Department of Medicinal Chemistry of the N.N. Vorozhtsov Institute of Organic Chemistry, Novosibirsk, Russia. The standard hydrocarbon mixture was obtained from Agilent Technologies (North Kingstown, RI, USA). The standard mixture of fatty acid methyl esters was obtained from Sigma-Aldrich (Steinheim, Germany). Syringol, Eugenol, Vanillin, Benzaldehyde, Benzoic acid, Phenol, 2,6-dimethoxy-4-(2-propenyl)- reagents obtained from Sigma- Aldrich were used as witnesses.

### 2.3. Metabolite Extraction, Derivatization, Detection by GC-MS, Data Processing and Annotation

Identification of extractive compounds: 1.0 g of a freshly collected material was extracted with 10 mL of methanol in a flask at room temperature for 48 h (stationary incubation with occasional stirring). The extract was passed through a Schott glass filter with a porosity of 10–16 μm. Next, 1 μL of the prepared extract of each sample was introduced into an injector of a gas chromatograph.

Preliminarily, the samples 1s and 7s were silylated according to a method from ref. [14], with modifications. In brief, the procedure included adding 0.3 mL each of pyridine, nitromethane, trimethylsilane, and hexamethyldisilazane to 10 mg of a dry residue of the extract. The glass containers with the reaction mixture were hermetically sealed and thoroughly shaken, then heated at 60 °C for 5 min. After cooling, the solution was passed through a Schott glass filter with a porosity of 10–16 µm, and 1 µL of the solution was introduced into the chromatograph.

One-dimensional GC-MS was carried out for quantitative analysis with subsequent statistical analysis of these data. Conditions for this analysis in one-dimensional mode were as follows: an Agilent Technologies 6890 chromatograph with mass spectrophotometric detector 5973; a capillary DB-1 J&W chromatographic column with an inner diameter of 0.25 mm and a length of 30 m; the carrier gas (helium) flow rate: 1.0 mL/min; sample injection heater temperature: 250 °C; thermostat temperature was programmed from 50 to 250 °C at a rate of 25 °C/min. A sample was introduced into the chromatographic column without flow splitting.

Retention indices (RIs) were determined by means of the chromatographic data from a mixture of standards (homologous linear alkanes (C8–C32)) and were calculated from retention times of peaks according to ref. [15].

The peaks were integrated both in the scanning mode with measurement of the total ion current in the mass range of 10–800 Da and in the mode of selective scanning of ions corresponding to the m/z value of a characteristic ion, which, as a rule, has the highest intensity (Q_m_). Peak areas of all extract components were calculated using the Xcalibur 2.0 software. For single-component peaks, the ratio R_Qm_ = S_t_/S_s_ for each of the compounds was calculated, where S_t_ is the area obtained in the total ion current mode, and S_s_ is the area determined in the selective ion mode with specific Q_m_. These R_Qm_ values were used as coefficients for determining component areas in multicomponent peaks.

Extract components were identified with the help of a built-in database of mass spectra—the NIST Mass Spectral Search Program for the NIST/EPA/NIH Mass Spectral Library Version 2.0a—as well as by comparison of the obtained RIs with RIs from the extended version of the same database [16]. In the data summary, we omitted chromatographic peaks belonging to substances of obvious anthropogenic origin: 2,5-di-tert-butyl-*p*-benzoquinone (RI = 1468), 3-tert-butyl-4-hydroxyanisole (1541), n-dodecyl acrylate (1676), dibutyl phthalate (1932), bis(2-ethylhexyl) fumarate (2207), and di-(2-ethylhexyl) phthalate (RI = 2511). Carbohydrates were disregarded if their chromatographic peaks were in the following ranges: for pentoses, RI = 1426 to 1515; for methyl hexoses formed during extraction with methanol, 1559 to 1752; and for sorbitol, 1826 to 2036. As a product of possible thermal dehydration of carbohydrates, a peak assigned to dihydro-3-methylene-5-methyl-2-furanone (1033) was also disregarded, which was present in almost all chromatograms. In this regard, there is no peak corresponding to this compound in chromatograms of extracts of *Crataegus pinnatifida* raw fruits, whereas it is quite noticeable in extracts if these fruits are roasted [17].

Values in the Table S1 are the peak areas of the compound associated with the reaction of the internal standard and assigned to the given metabolic pathway to the total peak areas of the identified compounds obtained on column DB-1 J&W, where the peak areas were calculated relative to those of the internal standard. The values in the table are expressed as a percentage.

In two-dimensional mode, the samples were analyzed on a Pegasus 4D GCxGC–TOF MS instrument with the following settings: injection, 1 mkl; pulsed split, 1:100, 250 °C; carrier gas (He) flow, 1.4 mL/min, corrected constant flow; column one, Rxi-5MS, 30 m × 0.25 mm i.d. × 0.25 µm coating (Restek, Centre County, PA, USA); column two, Rxi-17Sil MS, 1.75 m × 0.25 mm i.d. × 0.25 µm coating (Restek); temperature program, 50 °C (1 min), then at 5 °C/min to 150 °C, at 10 °C/min to 250 °C, and at 20 °C/min to 280 °C (and held there for 60 min); primary oven was kept at 5 °C higher than secondary oven; modulation, 8 s with temp. maintained at 15 °C above secondary oven; transfer line, 280 °C; ion source temp., 280 °C; mass range (m/z), 40–850.

Specificity (selectivity) was determined as follows: the solvent as a blank control was analyzed to confirm that there were no false peaks within the target range of retention times.

The obtained chromatograms were processed using the LECO ChromaTOF software, which automatically searches for and compares selected mass spectra with NIST electronic databases [16].

### 2.4. Statistical Methods

Hierarchical cluster analysis was used to visualize differences among the studied extracts of *S. aucuparia* bark collected during the winter and the summer periods. The compounds of these extracts, grouped according to metabolic pathways and their biosynthesis (see point 3.2), together with the total values of their percentages (Table 1 and Table 2) were considered the variables for the analysis of principal coordinate analysis (PCA) and unweighted paired group method with arithmetic mean (UPGMA). Eigenvalues and percentages of variation PC1 and PC2 were calculated by PCA. Similarity indices were estimated by the Bray–Curtis method. Multivariate analysis was performed in Past 3 v3.25 [18].

## 3. Results and Discussion

### 3.1. Identification of the Components of S. aucuparia subsp. sibirica Bark Extracts

A preliminary comparison between chromatograms from GC-MS analysis of an intact extract and of a trimethylsilylated extract from sample 1s (the letter s at the sample name indicates its summer origin, and w indicates its winter origin) showed that in the derivatized extract, there were no peaks corresponding to cyanogenic glycosides because of the decomposition of these compounds with the formation of the TMS derivative of mandelamide. Therefore, the silylation procedure was not used in all subsequent work.

**Figure 1:**
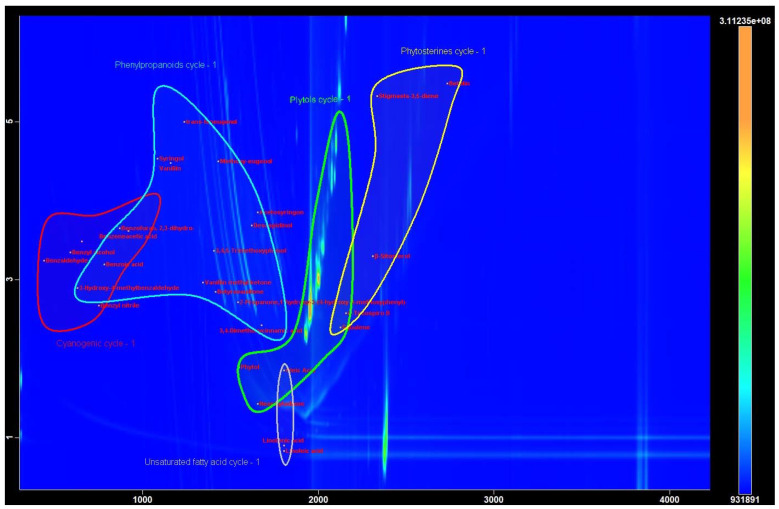
A contour plot of the GCxGC-TOF MS chromatogram of sample 1s (methanol extract of rowan *S. aucuparia* bark) containing peaks grouped by metabolic system classes: unsaturated fatty acids (purple outlining), products of the phenylpropanoid cycle (blue outlining), products of the cyanogenic pathway (red outlining), products of the phytol cycle (green outlining), and triterpenoids (yellow outlining).

In the extracts of bark samples collected from 16 plants in winter and in summer, 100 compounds were identified by GC-MS, including alkanes, alkenes, linear alcohols, fatty acids and their derivatives, phenols and their derivatives, prunasin and its precursors and derivative compounds, polyprenes and their derivatives, cyclic diterpenes, and phytosterols (Figure 1). Data on concentrations of these metabolites in extracts from summer and winter samples of rowan bark are given in Tables S2 and S3 (Supplementary Materials). According to area percentages, the overall proportion of fully or partially identified substances in the samples of rowan bark extracts was high: 95.48% to 98.14%.

**Figure 2:**
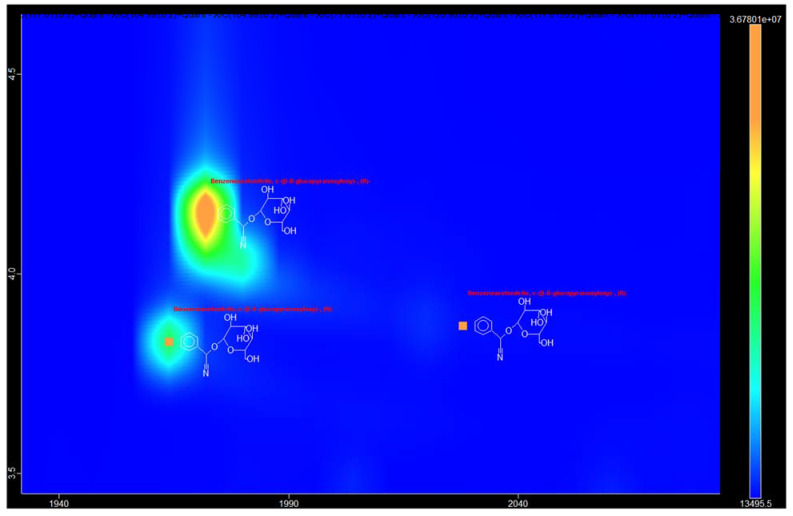
A contour plot of a portion of the GCxGC-TOF MS chromatogram of sample 7s (extract of *S. aucuparia* bark) containing peaks identified as prunasin. An exact assignment the peaks could not be performed.

In chromatograms of some samples (Figure 2), in the RI range from 2489 to 2650, two or three peaks having identical mass spectra were present, whose structure the system assigned to prunasin ((R)-α-(β-D-glucopyranosyloxy)-benzeneacetonitrile). It is known that the S-isomer of prunasin, sambunigrin, is found in a number of plants, for example, in representatives of the genus *Sambuca* [19]. The possibility of chromatographic separation of these isomers by GC-MS is stated in ref. [20], where it is suggested that the formation of sambunigrin is an artifact of the isolation procedure. Both prunasin and sambunigrin have been identified in bark extracts of another rowan species: *Sorbus cashmiriana* [21]. The third peak can be assigned to putative structure of an isomer having a modified configuration of the glycosidic bond.

### 3.2. Annotation of Identified Metabolites

Lately, such methods as hierarchical clustering and principal component analysis (PCA) have been widely used to statistically evaluate differences in the composition of metabolite mixtures. To apply these methods, source data can be grouped in various ways:

1. Processing of the dataset is carried out for all components (e.g., [22]).

2. Processing of the dataset is performed for those components, whose concentration is not lower than a certain value (e.g., [23]).

3. Processing of the dataset is carried out for components representing one class of compounds, for example, alkanes [24].

In the current work, all components are grouped by their biosynthesis pathway according to data from KEGG tables [25]. In particular, this approach will allow to minimize the influence of artifacts resulting from the processing of samples, for example, the formation of benzaldehyde from prunasin and sambunigrin during the disintegration of bark as well as the esterification of carboxylic acids by the solvent.

According to this method, most of the identified compounds could be assigned to six major metabolic pathways (summarized in Table 1 and Table 2).

No dependence of the location of a plant on the prevalence of individual metabolic pathways was found.

**Figure 3:**
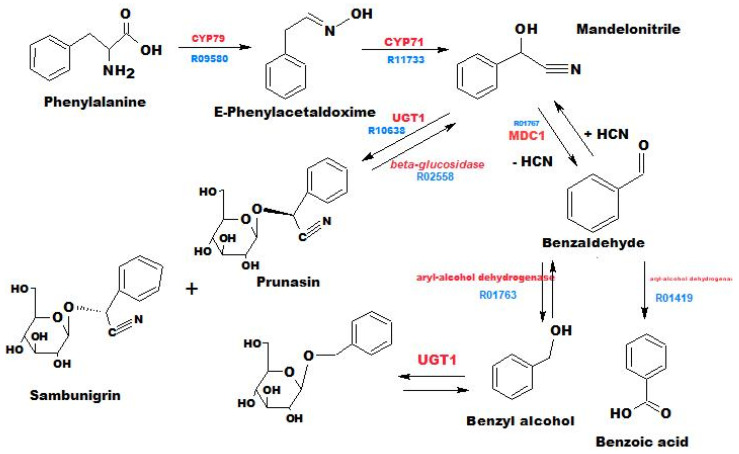
The general scheme of the pathway for the biosynthesis of cyanogenic compounds. The enzymes involved in the synthesis are marked in red, and metabolic pathways from the KEGG database are indicated in blue [25].

1. Pathway CC (Figure 3) is represented by benzaldehyde, benzyl alcohol, α-oxo benzeneacetonitrile, methyl ester of α-methoxy-benzeneacetic acid, benzyl nitrile, benzoic acid, mandelamide, benzyl β-D-glucoside, and prunasin.

The starting compound initiating this pathway is phenylalanine, which, as a result of intramolecular reactions involving enzymes CYP79 and CYP71, first transforms into E-phenylacetaldoxime, and then, after successive reactions, depending on the specificity of the enzymes involved, into one or both possible stereoisomers of mandelonitrile. After that, glucosylation of these isomers with the participation of enzyme UGT1 gives rise to prunasin and sambunigrin. The formation of benzyl-β-glucose, sometimes in substantial amounts, up to 41.18% (sample 6s), is explained by enzymatic glucosylation of benzyl alcohol, which arises during the reduction of benzaldehyde [26].

2. Pathway PhPC is represented by coumaran, 4-vinyl-2-methoxyphenol, 2,6-dimethoxy-phenol, eugenol, vanillin, trans-isoeugenol, vanillyl methyl ketone, 3’,5’-dimethoxyacetophenone, butyrovanillone, 2,6-dimethoxy-4-(2-propenyl)-phenol, 3,4,5-trimethoxyphenol, 4-hydroxy-3-methoxy-benzenepropanol, (E)-2,6-dimethoxy-4-(prop-1-en-1-yl)phenol, 1-hydroxy-3-(4-hydroxy-3-methoxyphenyl)-2-propanone, desaspidinol, 3,5-dimethoxy-4-hydroxyphenylacetic acid, acetosyringone, and 3,4-dimethoxycinnamic acid.

A large number of phenolic compounds—for example, eugenol, vanillin, trans-isoeugenol, vanillyl methyl ketone, butyrovanillone, 4-vinyl-2-methoxyphenol, 4-hydroxy-3-methoxy-benzenepropanol, 1-hydroxy-3-(4-hydroxy-3-methoxyphenyl)-2-propanone, 2,6-dimethoxy-phenol, acetosyringone, 2,6-dimethoxy-4-(2-propenyl)-phenol, (E)-2,6-dimethoxy-4-(prop-1-en-1-yl)phenol, and 3,5-dimethoxy-4-hydroxyphenylacetic acid—can be attributed to the presence of the polyphenolic polymer lignin in the bark. This set includes not only compounds found in the KEGG database of metabolic pathways but also substances structurally related to them. The total proportion of these compounds is relatively small and reaches a maximum of 13.12% in summer samples (9s) and 9.38% in winter samples (13w). Biosynthetic pathways for these compounds have been thoroughly studied and described (e.g., [27]).

**Figure 4:**
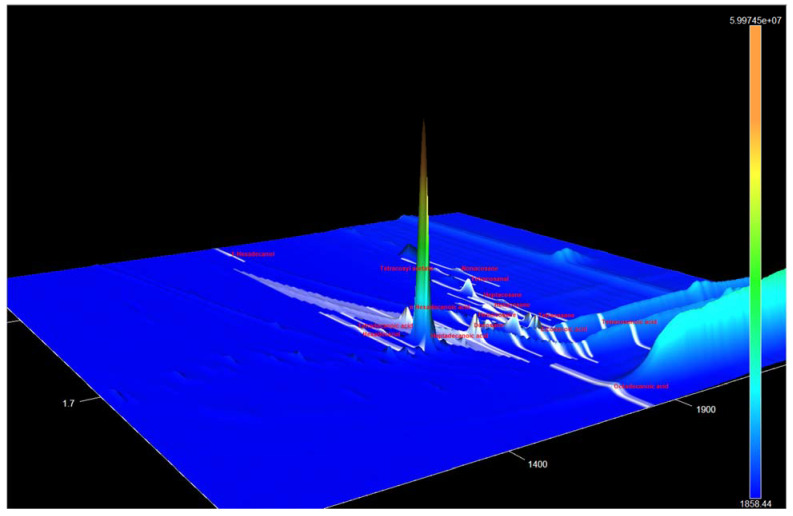
A contour plot of a portion of the GCxGC-TOF MS chromatogram (of a methanolic extract of *S. aucuparia* bark) containing peaks from the alkane metabolic pathway.

**Figure 5:**
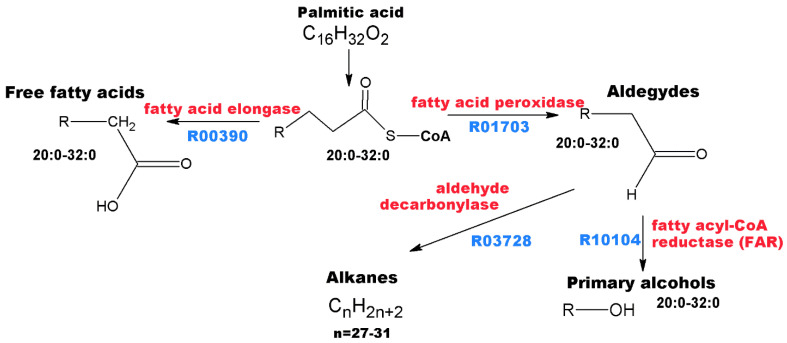
The general scheme of the alkane metabolic pathway according to refs. [25,28]. The enzymes participating in biosynthesis reactions are red, and metabolic pathways from the KEGG database are indicated in blue [25].

3. Pathway AC is represented by the largest number of compounds found in the extract samples (Figure 4), and these are substances from various chemical classes (Figure 5): alkanes (heneicosane, docosane, tricosane, tetracosane, pentacosane, hexacosane, heptacosane, and nonacosane), aliphatic alcohols of regular structure (1-hexadecanol, 1-octadecanol, 1-eicosanol, 1-heneicosanol, 1-docosanol, 1-tetracosanol and its acetate, and 1-hexacosanol), tetracosanal (an aldehyde), carboxylic acids (tetradecanoic acid, pentadecanoic acid, hexadecanoic acid, margarinic acid, stearic acid, eicosanoic acid, behenic acid, and lignoceric acid) and their derivatives methyl ester of hexadecanoic acid and N-ethyl octadecanamide.

This metabolic pathway is actually less pronounced in winter bark samples than in summer ones (no more than 4.76% in sample 8w versus 18.48% in sample 11s). Considering that sample 11s represents rowan table variety Nevezhenskaya, which contains a large amount of sugars in fruits, it is likely that the enhancement of the alkane pathway in the summer season correlates with the synthesis of carbohydrates in the plant.

**Figure 6:**
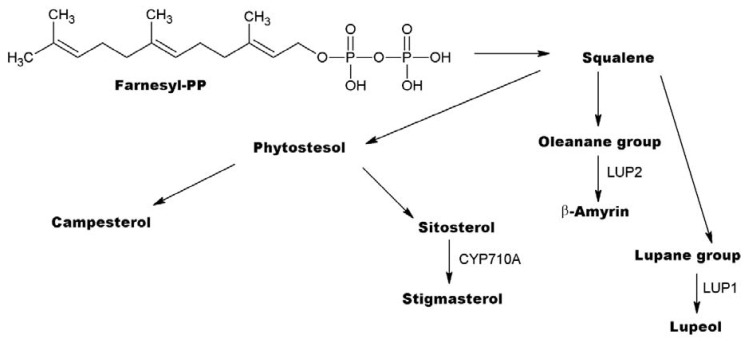
The general scheme of metabolic pathway PhSC [25].

4. Pathway UFAC: This is a minor pathway for the biosynthesis of unsaturated fatty acids and in the chromatograms of bark extracts gave peaks corresponding to palmitoleic acid, linoleic acid, linolenic acid, and oleic acid. In accordance with KEGG databases, this pathway proceeds as described in ref. [29].

5. Pathway PhSC (Figure 6) is represented by squalene, α-neooleana-3(5),12-diene, stigmastan-3,5,22-trien, stigmasta-3,5-diene, campesterol, β-sitosterol, β-amyrin, isofucosterol, lup-20(29)-en-3-one, α-amyrin, lupeol, stigmasta-3,5-dien-7-one, γ-sitostenone, lupeol acetate, betulinaldehyde, tillandsinine, betulinaldehyde acetate, betulin, and betulin acetate.

This set included not only compounds found in KEGG databases of metabolic pathways but also substances structurally related to them, that is, all cyclic triterpenes, together with their precursor squalene. According to data from KEGG databases, this transformation is mainly carried out through intermediate oxidation of squalene, in particular, with the formation of (all-E)-2,6,10,15,19,23-hexamethyl-1,6,10,14,18,22-tetracosahexaen-3-ol. The presence of this compound was detected only in the chromatogram of sample 9w obtained on the LECO Pegasus BT 4D system.

Cholesterol is a convenient seasonal marker because this compound was almost absent in winter samples.

In the chromatograms obtained by means of the Agilent Technologies 6890 system with mass-spectrophotometric detector 5973, the majority of samples in the RI range of 3442–3572 showed peaks with mass spectra reliably matching betulin, betulinaldehyde, and their acetates. Nonetheless, the first of these peaks has a significantly lower RI (3551) than that assigned to betulin in the NIST database (RI = 3740). At the same time, it is known that rowan bark contains not only betulin but also its isomer allobetulin, which has higher chromatographic mobility due to the replacement of the alcohol group by an ether one, without a change in spectral data [30].

In general, all these compounds are quite common in phytochemical mixtures and cannot be regarded as characteristic of this plant genus in any way (e.g., [31]).

**Figure 7:**
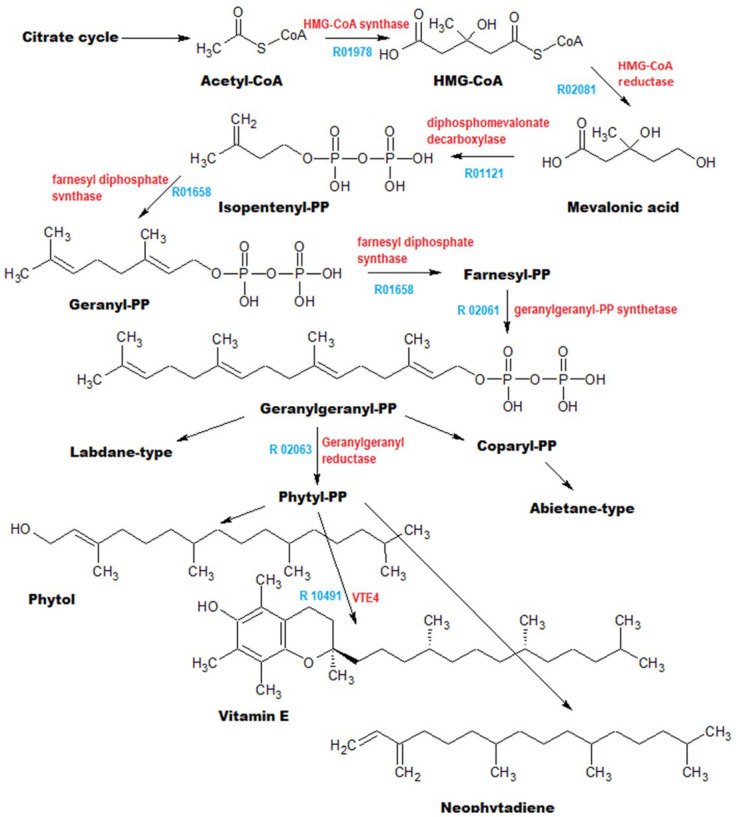
The general scheme of metabolic pathway PhC. The enzymes involved in biosynthetic reactions are marked in red, and metabolic pathways from the KEGG database are indicated in blue [25].

6. Pathway PhC. A different picture is observed when we described the compounds formed via the diterpene biosynthesis pathway (Figure 7). 

Although neophytadienes, phytol, dehydroabietic acid, 4,8,12,16-tetramethylheptadecan-4-olide, and vitamin E are also frequently found in phytochemical mixtures, the formation of tocospiro A and B has been reported in only one study [32]. In addition, chromatograms of almost all extracts contain a set of six peaks with RIs of 2604, 2787, 2801, 2810, 2831, and 2914 (in the Agilent Technologies 6890 system with mass-spectrophotometric detector 5973), and the total content of these peaks reached 45.86%. During identification of structures of these compounds by comparison with NIST databases, either betulin or lupeol were at the top of the list, with a reliability of 40–60%; however, both of these triterpenoid phytosterols are present in the analyzed extracts and have much higher RIs. GC-MS data on these compounds are presented in Table 3. 

**Figure 8:**
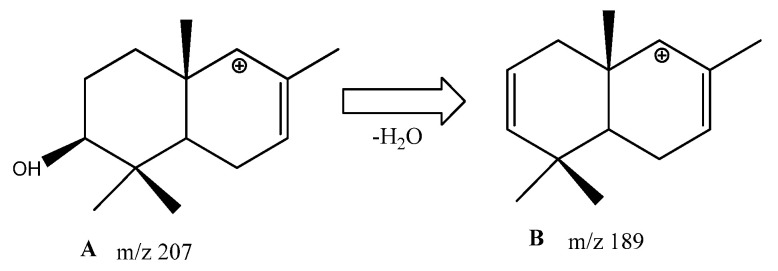
Structure of ions—seen in the mass spectra of components of bark extracts from *S. aucuparia*—corresponding to characteristic peaks with *m*/*z* = 207 and 189 according to [34].

Since these compounds have not yet been isolated in pure form and have not been identified, we can only guess their likely structure based on the presence of characteristic peaks with m/z = 207 and 189, which are shared with lupeol and betulin. These peaks, according to [33], correspond to structures of ions A and B (Figure 8):

In the reaction mixture after silylation of extract sample 1s, among others, a peak was found, presumably matching β-levantenolide (labd-13-en-15-oic acid, 8,12-epoxy-12-hydroxy-, γ-lactone, (12S)-) with an RI of 3043.8. Given that this peak is absent in the chromatograms of intact extracts, it is possible that this compound is produced by the reaction of the silylating mixture with one of the above-mentioned substances. In any case, this is an additional confirmation of the idea that these compounds possess labdane structure.

### 3.3. Statistical Analysis

**Figure 9:**
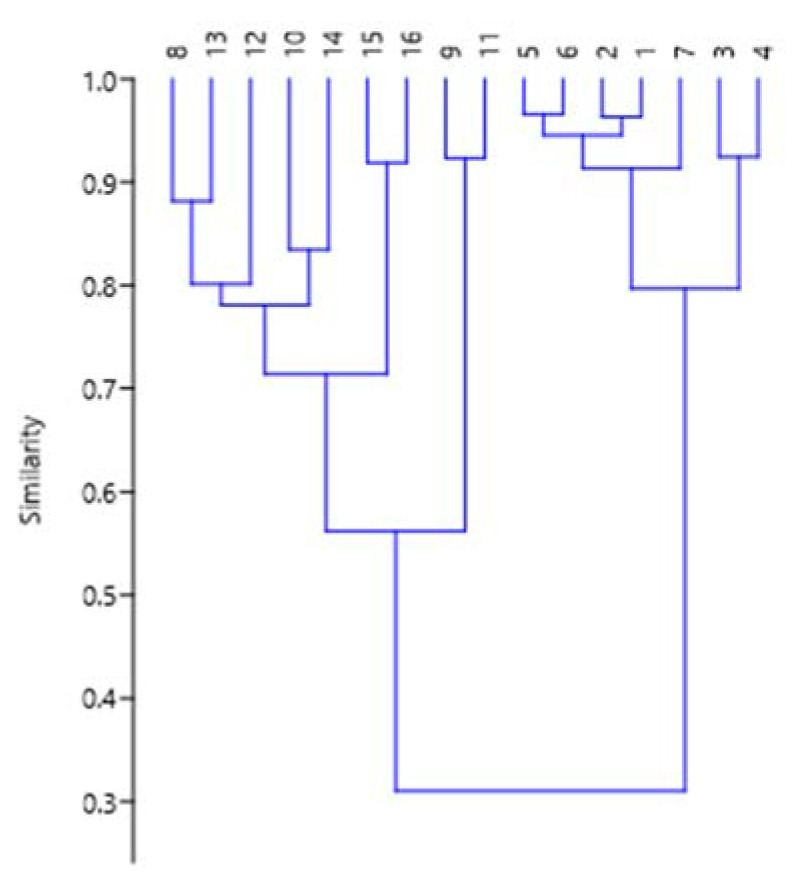
The hierarchical clustering dendrogram of components of extracts from winter *S. aucuparia* bark, as classified by their biosynthesis pathways. The vertical axis shows similarity indices, and the horizontal axis presents ID numbers of samples.

Hierarchical cluster analysis was chosen to visualize differences among the studied extracts of *S. aucuparia* bark collected during the winter period. Figure 9 shows a dendrogram of lipophilic components of the extracts as determined by UPGMA (unweighted pair group method with arithmetic mean).

**Figure 10:**
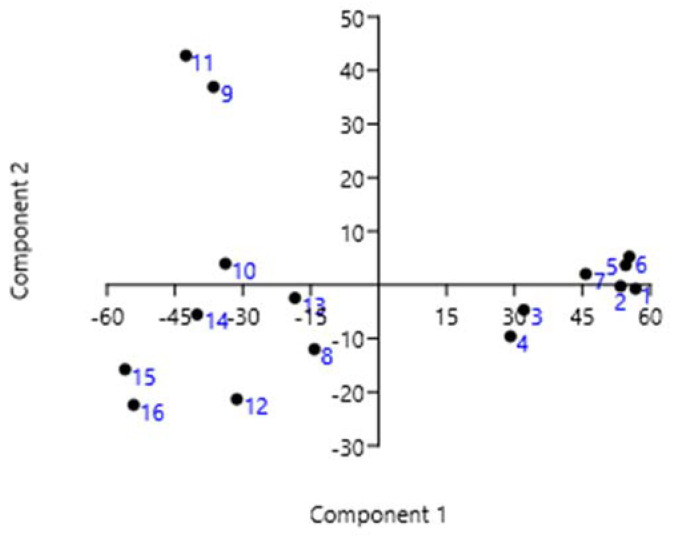
Biplots of PCA of compounds in winter extracts of rowan bark. The two dimensions together explain 98% of the data.

The cophenetic correlation coefficient was high (0.9154), meaning that the dendrogram accurately reflects similarities between observations. The dendrogram shows grouping into three main branches. Samples 1w–7w are grouped on one side, where metabolites synthesized within the phenylalanine pathway predominate (>70%), with a similarity index of 0.32, as estimated by the Bray–Curtis method. On the left side, extracts are grouped in which phytosterols dominate over representatives of the phenylalanine pathway; furthermore, in two samples (9w and 11w) with a similarity index of 0.57, the content of phytosterols does not exceed one third, but there is a substantial proportion of compounds synthesized via the phytol pathway. PCA [13] illustrated by the graph in Figure 10 confirmed this observation. This approach also indicated that almost the whole distribution of metabolites as follows from the values of the percentages of variability by the first two principal components: PC1 and PC2 (85.26% and 13.70%). Eigenvalues are 485.532 and 113.753, respectively.

**Figure 11:**
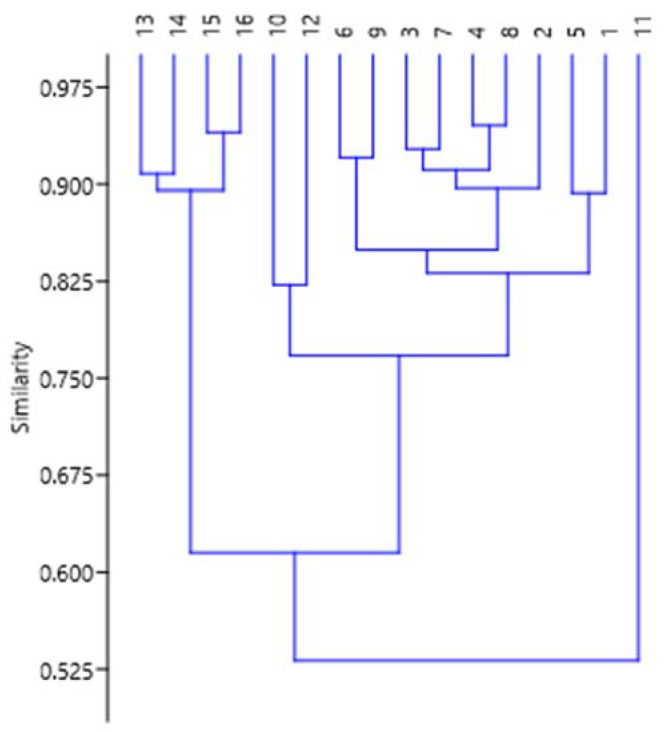
The hierarchical clustering dendrogram for components of extracts from summer *S. aucuparia* bark, as classified by their biosynthesis pathways. The Y-axis shows similarity indices, and the X-axis shows ID numbers of samples.

The dendrogram for lipophilic components of the extracts of samples collected in summer from the same trees (Figure 11) differs only slightly from the dendrogram described above. Sample 11s is still at its isolated position, whereas sample 8s migrated to another branch of the tree. The cophenetic correlation coefficient turned out to be slightly lower (0.8938) in comparison with the winter one. The similarity index for the two main clusters is only 0.61, meaning that differences in metabolic pathways are much less pronounced in summer samples.

**Figure 12:**
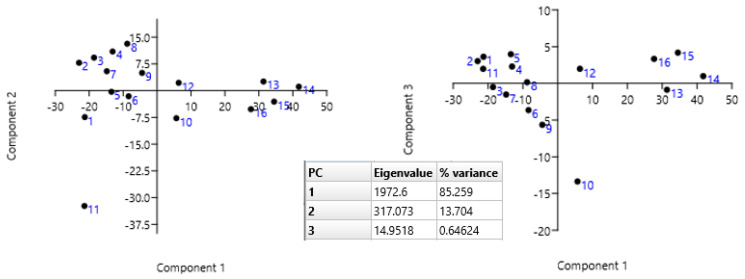
(Left, right). Biplots of PCA of compounds in summer extracts from rowan bark. The inner table contains the Eigenvalues and the percentage of variability for the first three principal components.

Furthermore, according to Figure 12, in the cluster that combines trees with the predominance of the phenylalanine metabolic pathway, samples 13s–16s retained their properties. PCA provided additional information. First, there was a reversal of some positive and negative loadings along the PC1 axis. Samples 13s–16s, which show predominance of phytosterols, formed a distinct cluster. In samples located in the region of negative loadings, the amounts of products of the phenylalanine and phytosterol cycles are comparable, but without the prevalence of the former. The negative PC2 value, explained by the influence of elevated levels of phenolic and phytol compounds, separates sample 11s from both clusters in Figure 12 left. The first two principal components together account for 94.86% of the total variance among the bark extract samples. The negative PC3 value, explained by the influence of increased concentrations of phenolic compounds, separates sample 10s from both clusters in Figure 12 right. Overall, it can be concluded that in winter, the accumulation of products of the phenylalanine cycle is more active.

The PCA that was calculated from the data matrix of such contributions of metabolic pathways in summer extract samples yielded two clusters of obvious chemotaxonomic similarity (Figure 12 left). Here, positive PC1 values separated the chemotype with a dominant contribution of the cyanogenic pathway from the chemotype featuring the predominant phytosterol pathway explained by relatively high percentage abundance of prunasin, sambunigrin, and benzaldehyde. A similar conclusion about the ratio of cyanogenic and phytosterol pathways for winter samples is confirmed by the PC1-PC6 loads for the six metabolic pathways in winter rowan bark samples presented in Table 4.

It should be noted that similar polymorphism in terms of the synthesis of cyanogenic compounds within the same population of a species has been described previously [20]. Namely, among 60 specimens of *Eucalyptus nobilis* seedlings, 22 specimens proved to be acyanogenic, and among them, 17 specimens did not contain prunasin, and in five specimens, there was prunasin but no β-glucosidase.

**Figure 13:**
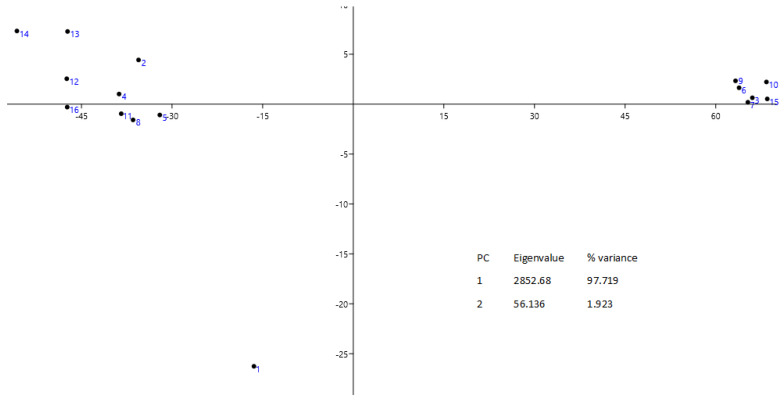
Biplots of PCA of compounds (from summer extracts of *S. aucuparia* bark) affiliated with the phytosteroid biosynthesis pathway.

PCA in Figure 13 shows that the studied bark samples manifested polymorphism of phytosteroid biosynthesis. By means of PCA, we estimated the distribution of summer extracts of *S. aucuparia* bark within one metabolic pathway. The distribution is 97.7% attributable to PC1 and is almost bipolar; on the left side of the distribution, there are samples with a prevailing content of lupeol, and on the right, β-sitosterol dominates. Sample No. 1 is somewhat separated from these clusters, and its chromatogram in the range RT 42,476–45,522 contains additional peaks with a total intensity of 20%, which the AMDIS software also identified as lupeol.

It is known that the bark of rowan species of East Asian origin does not contain flavone compounds, whereas in bark extracts from *Sorbus* of Northern European origin, some authors have detected derivatives of quercetin and of 3-β-glucoside of 3,5,7,4’-tetrahydroxy-8-methoxy-flavone [35]. Since no flavone compounds were reliably detected in chromatograms of our samples, it can be assumed that our rowan specimens have more in common with the East Asian population than with the Northern European one.

### 3.4. Pharmacological Significance of Annotated Compounds

The genus *Sorbus* L. is an ethnopharmacologically important but underappreciated set of various species. Remedies prepared from its leaves, bark, or fruits have long been used in folk medicine [36]; however, the review just cited shows that in the case of *S. aucuparia*, folk medicine practitioners have mainly used fruit extracts, leaves, and flowers of this plant. Studies on pharmacological effects of extracts from *S. aucuparia* bark are few and far between. For instance, Sak et al. [37] report that tea made from *S. aucuparia* bark has been employed in Estonia to treat cancer. Somewhat more is known about pharmacological applications of extracts from the bark of related rowan species: *S. decora*, *S. americana*, *S. commixta*, *S. cashmiriana,* and *S. pohuashanensis*. These extracts possess antidiabetic, vasorelaxant, hypoglycemic, anti-inflammatory, anticancer, antiarthritic, and other medicinal properties, data on which are summarized in reviews [36,38].

One of the potential sources of biological activities in bark extracts from *S. aucuparia* is phytol, which can act as an excellent immunostimulant having antioxidant, anti-inflammatory, and antiallergic properties [39]. α-Tocopherol, also known as vitamin E, is another important secondary metabolite found in these bark extracts and has one of the strongest biological effects in most biological systems. Due to its fat solubility, α-tocopherol has the unique function of protecting cell membranes (which are composed of fatty acids) from damage by reactive oxygen species (ROS); moreover, it takes part in photosynthesis [40]. Lupenone and lupeol inhibit protein tyrosine phosphatase 1B (PTP1B) [41], whereas α- and β-amyrins significantly attenuate cerulein-induced upregulation of tumor necrosis factor (TNF), interleukin 6, lipase, amylase, myeloperoxidase (MPO), and thiobarbituric acid reactive substances (TBARS). Moreover, the above compounds significantly suppress pancreatic edema, infiltration by inflammatory cells, acinar-cell necrosis, and the expression of TNF and inducible nitric oxide synthase [42].

Of note, β-sitosterol glucoside exerts anti-inflammatory actions both in vitro and in vivo, reduces the production of nitric oxide, PGE-2, TNF, IL-1b, and IL-6 (together with the expression of iNOS and COX- 2), and suppresses the activation of NF-kB in LPS-stimulated RAW 264.7 cells [43].

On the other hand, practical application of formulations from the bark of S. aucuparia is limited by the presence of cyanogenic glycosides prunasin and sambunigrin in it. They can be hydrolyzed by β-glucosidase into unstable cyanohydrin, which degrades into toxic hydrocyanic acid. One gram of prunasin can release 91.5 mg of HCN. As a consequence, HCN from rowan organs—when forming at levels exceeding 2–3 mg/(kg of human body weight)—can cause respiratory arrest and even death [44]. Therefore, cyanogenic glycosides that are present in rowan bark may perform a protective function for the plant, by preventing damage by ruminants [45] and insects [46].

## 4. Conclusions

Thus, in the present work, it was demonstrated that within one population of *S. aucuparia* under similar growth conditions, the profile of lipophilic metabolites in the bark differs noticeably among these trees. Our examination of the concentrations of these metabolites by hierarchical cluster analysis and PCA uncovered the presence of at least two chemotaxonomic subpopulations. Seasonal differences in the profile of metabolites were revealed too. The usefulness of classification of metabolites by biosynthetic pathways was proven for this kind of analysis. It was found that the species affiliation is not the only determinant of the metabolite profile, and this observation may be helpful for possible practical use of these plants. The newly developed methodology may facilitate the selection of these plants by traditional techniques and genetic engineering manipulations of Sorbus taxa containing certain biologically active substances.

## Figures and Tables

**Table 1:** Classification of the metabolites (identified by GC-MS analysis of summer extracts of *S. aucuparia* bark) into six metabolic pathways. Abbreviations: CC, cyanogenic cycle; PhPC, phenylpropanoid cycle; PhC, phytol cycle; PhSC, phytosterol cycle; UFAC, unsaturated fatty acid cycle; and AC, alkane cycle. The values in the table are the sum of the content values of the components from Table S1 relating to the relevant metabolic pathways. The values in the table are expressed as a percentage. For this table and other illustrations below, ID numbers of samples can be looked up in Table S1 (Supplementary Materials).

	CC	PhPC	PhC	PhSC	UFAC	AC
1	29.99	2.02	10.28	29.97	6.96	15.04
2	42.91	1.46	7.55	35.88	0.56	10.19
3	41	3.3	5.83	39.7	2.15	6.68
4	39.32	1.51	4.15	45.17	0.34	9.38
5	31.69	1.8	10.17	40.22	0.86	12.96
6	27.88	7.29	12.38	43.63	1.06	5.92
7	35.58	5.5	4.06	40.86	2.64	10.03
8	38.05	2.09	2.64	49.23	0.66	6.26
9	28.9	7.78	4.53	48.54	0.68	5.17
10	13.29	16.52	5.84	50.84	3.33	6.48
11	13.53	7.56	26.15	20.18	7.67	18.84
12	21.5	1.85	5.27	56.95	2.23	10.39
13	7.61	1.84	6.57	77.24	0.15	3.04
14	0.78	0.27	6.42	85.26	0.06	4.18
15	2.19	0.22	7.08	77.99	0.04	11.29
16	4.21	1.59	7.41	71.21	0.04	12.54

**Table 2:** Classification of metabolites (identified by GC-MS analysis of winter extracts of *S. aucuparia* bark) into six metabolic pathways. Abbreviations: CC, cyanogenic cycle; PhPC, phenylpropanoid cycle; PhC, phytol cycle; PhSC, phytosterol cycle; UFAC, unsaturated fatty acid cycle; and AC, alkane cycle. The values in the table are the sum of the content values of the components from Table S1 relating to the relevant metabolic pathways. The values in the table are expressed as a percentage.

	CC	PhPC	PhC	PhSC	UFAC	AC
1	85.87	1.72	3.27	4.35	0.45	3.71
2	84.13	0.94	5.35	6.98	0.34	3.73
3	69.21	1.53	8.12	22.95	0.09	2.94
4	64.52	1.23	1.7	24.09	0.4	0.85
5	84.3	1.79	8.17	4.04	0.62	3.12
6	83.7	3.45	8.18	1.69	0.54	0.96
7	77.76	2.72	9.09	10.13	0.66	2.95
8	31.52	1.68	11.17	50.69	0.72	4.76
9	5.38	2.38	55.26	34.8	0.05	1.31
10	13.95	1.89	29.13	53.4	0.04	0.88
11	0.12	0.01	61.93	35.43	0.04	1.07
12	21.07	1.2	9.1	67.54	0.06	0.57
13	28.41	9.38	21.54	49.84	0.42	1.93
14	2.49	0	15.26	53.65	0	1.97
15	0.19	0.03	19.18	77.7	0	2.98
16	0.12	0	11.19	77.31	0	10.71

**Table 3:** Fragmentation patterns of diterpenic compounds in the GC-MS analysis of sample 6s. Boldfaced mass and relative abundance indicate a base peak.

RI	Mol. Peak (m/z)	Main Fragments Observed, m/z (Relative Abundance, %)
2604	318	318 (36), **207 (100)**, 190 (43), 189 (79), 135 (49), 121 (35), 109 (34), 108 (51), 95 (62), 81 (36)
2787	334	248 (45), 247 (54), **207 (100)**, 189 (36), 135 (47), 121 (44), 107 (42), 95 (43), 81 (40), 55 (34)
2801	332	332 (22), 314 (17), 299 (22), **207 (100)**, 206 (32), 192 (15), 189 (18), 135 (20), 124 (35), 97 (68)
2804	334	208 (45), **207 (100)**, 190 (40), 189 (45), 135 (32), 111 (22), 108 (51), 107 (24), 97 (76), 55 (24)
2831	332	**207 (100)**, 206 (28), 191 (25), 189 (26), 135 (28), 124 (28), 123 (23), 121 (22), 107 (22), 97 (92)
2914	358	317 (74), **299 (100)**, 207 (72), 191 (51), 189 (52), 135 (37), 109 (38), 95 (57), 81 (33), 43 (46)

**Table 4:** Loadings in principal components PC1–PC6 for the six metabolic pathways in samples 1w–16w.

	PC 1	PC 2	PC 3	PC 4	PC 5	PC 6
CC	805.84	−162.3	545.64	118.75	−111.62	−1.4901
PhPrC	8.7344	7.693	267.77	−515.57	812.86	−40.083
PHC	−225.62	803.7	522.41	155.08	−78.748	3.1565
PStC	−547.35	−569.45	593.17	93.287	−124.76	3.9015
UAC	4.3085	−1.1393	6.4272	−8.6889	41.601	999.07
AC	−6.1579	−58.249	−76.042	829.01	550.73	−15.273

## Data Availability

Data recorded in the current study are available in all Tables and Figures of the manuscript and of the Supplementary Materials.

## Supplementary Materials

The following supporting information can be downloaded at: https://www.mdpi.com/article/10.3390/metabo13060768/s1, Table S1. The ID numbers of samples and geographical location of the collected samples; Table S2. Compounds identified in extracts from *S.aucuparia* bark by GC/MS (summer gathering samples); Table S3. Compounds identified in extracts from *S.aucuparia* bark by GC/MS (winter gathering samples).

## References

[1] Polatoğlu K. (2013). “Chemotypes”—A Fact that should not be Ignored in Natural Product Studies. Nat. Prod. J..

[2] Martucci M.E.P., Vos R.C.H.D., Carollo C.A., Gobbo-Neto L. (2014). Metabolomics as a Potential Chemotaxonomical Tool: Application in the Genus Vernonia Schreb. PLoS ONE.

[3] Aldasoro J.J., Aedo C., Navarro C., Garmendia F.M. (1998). The genus Sorbus (Maloideae, Rosaceae) in Europe and in North Africa: Morphological analysis and systematics. Syst. Bot..

[4] Gabrielian E.T. (1978). Ryabiny (Sorbus L.) Zapadnoi Azii i Gimalayev [Rowans (Sorbus L.) of Western Asia and Himalayas].

[5] McAllister H. (2005). The Genus Sorbus: Mountain Ash and Other Rowans.

[6] Robertson A., Rich T.C.G., Allen A.M., Houston L., Roberts C., Bridle J.R., Harris S.A., Hiscock S.J. (2010). Hybridization and polyploidy as drivers of continuing evolution and speciation in Sorbus. Mol. Ecol..

[7] Caudullo G., Welk E., San-Miguel-Ayanz J. (2017). Chorological maps for the main European woody species. Data Brief.

[8] Polozhij A.V., Malyschev L.I. (2004). Rosaceae. Flora of Siberia.

[9] Shaulo D.N., Drachev N.S., Kuzmin I.V. (2009). Introgressive hybridization of Sorbus (Rosaceae) in Tyumen region boreal forests. Vestnik Tyumenskogo Gosudarstvennogo Universiteta Ekologiya Prirodopol’zovanie.

[10] Asbaganov S.V. Leaf morphological variation among populations of mountain ash across Russia. Proceedings of the Problemy Izucheniya Rastitel’nogo Pokrova Sibiri, Materialy V International Scientific Conference.

[11] Rengarten G.A., Sorokopudov V.N. (2019). Selection of rows as a decorative culture in Russia and in European countries. Vestnik KrasGAU Agron..

[12] Goncharov N.P., Savel’ev N.I. (2015). On the 160th anniversary of Ivan V. Michurin’s birth. Vavilovskii Zhurnal Genetiki Selektsii Vavilov J. Genet. Breed..

[13] State Register for Selection Achievements Admitted for Usage (National List) (2021). “Plant Varieties” (Official Publication).

[14] Kadam S.T., Kim S.S. (2010). Catalyst-free silylation of alcohols and phenols by promoting HMDS in CH_3_NO_2_ as solvent. Green Chem..

[15] Van Den Dool H., Kratz P.D. (1963). A Generalization of the Retention Index System Including Linear Temperature Programmed Gas-Liquid Partition Chromatography. J. Chromatogr. A.

[16] https://webbook.nist.gov/chemistry/gc-ri/.

[17] Zhong L., Wang Y., Peng W., Liu Y., Wan J., Yang S., Li L., Wu C., Zhou X. (2015). Headspace Solid-Phase Microextraction Coupled with Gas Chromatography-Mass Spectrometric Analysis of Volatile Components of Raw and Stir-Fried Fruit of C. pinnatifida (FCP). Trop. J. Pharm. Res..

[18] Hammer Ø.H., Harper D.A.T., Ryan P.D. (2001). Past: Paleontological Statistics Software Package for Education and Data Analysis. Palaeontol. Electron..

[19] Buhrmester R.A., Ebinger J.E., Seigler D.S. (2000). Sambunigrin and cyanogenic variability in populations of *Sambucus canadensis* L. (Caprifoliaceae). Biochem. Syst. Ecol..

[20] Gleadow R.M., Vecchies A.C., Woodrow I.E. (2003). Cyanogenic *Eucalyptus nobilis* is polymorphic for both prunasin and specific β-glucosidases. Phytochemistry.

[21] Rizvi T.S., Ali L., Shaheen F. (2013). Phytochemical Investigations on Sorbus Cashmiriana: Isolation, Structure Elucidation and Biological Evaluation of the Chemical Constituents.

[22] Pastor K., Ačanski M., Vujić Đ., Jovanović Đ., Wienkoop S. (2016). Authentication of Cereal Flours by Multivariate Analysis of GC–MS Data. Chromatographia.

[23] He X., Wang S., Shi J., Sun Z., Lei Z., Yin Z., Qian Z., Tang H., Xie H. (2018). Genotypic and Environmental Effects on the Volatile Chemotype of *Valeriana jatamansi* Jones. Front. Plant Sci..

[24] Maffei M. (1996). Chemotaxonomic significance of leaf wax alkanes in the gramineae. Biochem. Syst. Ecol..

[25] Kegg Pathway Database. https://www.kegg.jp/kegg/pathway.html.

[26] Sánchez-Pérez R., Jørgensen K., Olsen C.E., Dicenta F., Møller B.L. (2008). Bitterness in almonds. Plant Physiol..

[27] Biała W., Jasiński M. (2018). The Phenylpropanoid Case—It Is Transport That Matters. Front. Plant Sci..

[28] Kunst L., Samuels A.L. (2003). Biosynthesis and secretion of plant cuticular wax. Prog. Lipid Res..

[29] He M., Qin C.-X., Wang X., Ding N.-Z. (2020). Plant Unsaturated Fatty Acids: Biosynthesis and Regulation. Front. Plant Sci..

[30] Lawrie W., McLean J., Taylor G.R. (1960). Triterpenoids in the bark of mountain ash (*Sorbus aucuparia* L.). J. Chem. Soc..

[31] Radulović N.S., Đorđević N.D. (2011). Steroids from poison hemlock (*Conium maculatum* L.): A GC–MS analysis. J. Serb. Chem. Soc..

[32] Chiang Y.-M., Kuo Y.-H. (2003). Two novel α-tocopheroids from the aerial roots of *Ficus microcarpa*. Tetrahedron Lett..

[33] De Carvalho T.C., Polizeli A.M., Turatti I.C.C., Severiano M.E., de Carvalho C.E., Ambrósio S.R., Crotti A.E.M., de Figueiredo U.S., Vieira P.C., Furtado N.A.J.C. (2010). Screening of Filamentous Fungi to Identify Biocatalysts for Lupeol Biotransformation. Molecules.

[34] Varmuza K., Filzmoser P. (2008). Introduction to Multivariate Statistical Analysis in Chemometrics.

[35] Challice J.S. (1973). Phenolic Compounds of The Subfamily Pomoideae: A Chemotaxonomic Survey. Phytochemistry.

[36] Sołtys A., Galanty A., Podolak I. (2020). Ethnopharmacologically important but underestimated genus Sorbus: A comprehensive review. Phytochem. Rev..

[37] Sak K., Jürisoo K., Raal A. (2014). Estonian folk traditional experiences on natural anticancer remedies: From past to the future. Pharm. Biol..

[38] Sarv V., Venskutonis P.R., Bhat R. (2020). The *Sorbus* spp.—Underutilised Plants for Foods and Nutraceuticals: Review on Polyphenolic Phytochemicals and Antioxidant Potential. Antioxidants.

[39] Santos C.C., Salvadori M.S., Mota V.G., Costa L.M., De Almeida A.A.C., De Oliveira G.A.L., Costa J.P., de Sousa D.P., De Freitas R.M., De Almeida R.N. (2013). Antinociceptive and antioxidant activities of phytol in vivo and in vitro models. J. Neurosci..

[40] Brigelius-Flohé R., Traber M.G. (1999). Vitamin E: Function and metabolism. FASEB J..

[41] Na M., Kim B.Y., Osada H., Ahn J.H. (2009). Inhibition of protein tyrosine phosphatase 1B by lupeol and lupenone isolated from Sorbus commixta. J. Enzym. Inhib. Med. Chem..

[42] Melo C.M., Morais T.C., Tomé A.R. (2011). Anti-inflammatory effect of α,β-amyrin, a triterpene from *Protium heptaphyllum*, on cerulein-induced acute pancreatitis in mice. Inflamm. Res..

[43] Yang G., An H.-J. (2014). β-sitosteryl-3-O-β-glucopyranoside isolated from the bark of *Sorbus commixta* ameliorates pro-inflammatory mediators in RAW 264.7 macrophages. Immunopharmacol. Immunotoxicol..

[44] Alexander J., Autrup H., Bard D., Benford D., Carere A., Costa L.G., Verger P. (2007). Opinion of the scientific panel on contaminants in the food chain on a request from the Commission related to cyanogenic compounds as undesirable substances in animal feed. EFSA J..

[45] Cheeke P.R. (1989). Toxicants of Plant Origin: Glycosides.

[46] Patton C.A., Ranney T.G., Burton J.B. (1997). Natural pest resistance of Prunus taxa to feeding by adult Japanese beetles: Role of endogenous allelochemicals in host resistance. J. Am. Hortic. Sci..

