# Supplementary material for: A GC-MS Chemotaxonomic Study on Lipophilic Compounds in the Bark of S. aucuparia subsp. sibirica Trees from the Population Growing in Akademgorodok, Novosibirsk (Russia)
Source: Metabolites. 2023 Jun 19;13(6):768. doi: 10.3390/metabo13060768 (PMC10301433; doi:10.3390/metabo13060768)
Supplement: Supplementary file 1 [file metabolites-13-00768-s001.zip › metabolites-2389929-supplementary.pdf]

Table S1. The ID numbers of samples and geographical location of the collected samples.

1.	coordinates: 54.84840, 83.12129; red-orange fruits, moderately bitter; collected 13.07.2022 and 11.02.2022.
2.	coordinates: 54.84024, 83.11121; orange fruits, moderately bitter; collected 02.07.2022 and 14.02.2022.
3.	coordinates: 54.84351, 83.11314; orange fruits, non-bitter collected 02.07.2022 and 17.02.2022.
4.	coordinates: 54.84797, 83.12050; orange fruits, moderately bitter; collected 13.06.2021 and 27.01.2022.
5.	coordinates: 54.83514, 83.11054; orange fruits, moderately bitter; collected 02.07.2022 and 14.02.2022.
6.	coordinates: 54.84962, 83.10374; orange fruits, non-bitter collected 02.07.2022 and 11.01.2022.
7.	coordinates: 54.849367, 83.104057; orange fruits, non-bitter collected 02.07.2021 and 29.01.2022.
8.	coordinates: 54.84828, 83.12216; orange fruits, non-bitter collected 13.06.2021 and 27.01.2022.
9.	coordinates: 54.84440, 83.11603; orange fruits, non-bitter collected 02.07.2021 and 12.02.2022.
10.	coordinates: 54.848854, 83.103684; orange fruits, non-bitter collected 30.05.2021 and 17.03.2022.
11.	coordinates: 54.84966, 83.1068; orange fruits, moderately bitter; collected 16.09.2020 and 12.02.2022.
12.	coordinates: 54.84958, 83.10378; orange fruits, bitter; collected 11.08.2021 and 27.01.2022.
13.	coordinates: 54.849388, 83.104022; orange fruits, bitter; collected 13.06.2021 and 12.02.2022.
14.	coordinates: 54.84545, 83.10626; orange fruits, bitter; collected 02.07.2021 and 12.02.2022.
15.	coordinates: 54.84768, 83.11914; orange fruits, moderately bitter; collected 02.07.2021 and 17.03.2022.
16.	coordinates: 54.84788; 83.12018 red-orange fruits, moderately bitter; collected 27.05.22 and 14.02.22

Table S2. Compounds identified in extracts from *S.aucuparia* bark by GC/MS (summer gathering samples)

#	Compound	RI	RI_{lit}	Q_m	1s	2s	3s	4s	5s	6s	7s	8s	9s	10s	11s	12s	13s	14s	15s	16s
001	Benzaldehyde	957	965	106	11,49	0,49	33,14	0,73	0,31	8,72	33,83	0,56	8,74	5,06	13,53	0,09	0,35	0,05	0,25	2,67
002	Benzyl alcohol	1019	1026	108	0,14	0,00	0,01	0,03	0,00	1,96	0,01	0,01	0,21	0,08	0,00	0,01	0,03	0,01	0,19	0,06
003	Benzeneacetonitrile, α-oxo-	1077		131	0,05	0,10	0,01	0,08	0,06	0,02	0,00	0,16	0,21	0,21	0,21	0,02	0,00	0,01	0,35	0,06
004	Benzeneacetic acid, α-methoxy-, methyl ester	1088	-	121	0,36	0,00	0,00	0,00	0,00	8,40	0,00	0	0,07	0	0,00	0	0,13	0,00	0,00	0,05
005	Benzeneacetonitrile	1111	1103	117	1,07	0,10	0,04	0,13	0,00	1,50	0,02	0,11	0,07	0,25	0,15	0,02	0,01	0,01	0,39	0,05
006	Benzoic acid	1164	1159	122	0,51	0,41	0,06	1,50	0,34	0,59	0,01	0,45	1,77	1,64	0,60	0,09	0,03	0,01	0,75	0,51
007	Coumaran	1192	1188	120	0,15	0,06	0,06	0,01	0,06	0,00	0,01	0,01	0,49	1,88	0,01	0,02	0,00	0,01	0,44	0,21
008	4-Vinyl-2-mehoxyphenol	1298	1317	135	0,03	0,02	0,40	0,03	0,12	0,31	0,02	0,02	0,80	0,12	0,03	0,04	0,01	0,01	0,11	0,19

009	Phenol, 2,6-dimethoxy-	1320	1320	154	0,15	0,50	0,04	0,03	0,02	0,12	0,02	0,01	0,58	0	0,03	0,02	0,01	0,01	0,01	0,19
010	Eugenol	1341	1335	164	0,03	0,05	0,37	0,06	0,54	0,18	0,23	0,04	2,26	0	0,05	0,20	0,01	0,01	0,24	0,71
011	Vanillin	1367	1357	152	0,03	0,04	0,10	0,04	0,06	0,17	0,06	0,01	0,03	0,13	0,00	0,50	0,01	0,03	0,03	0,58
012	trans-Isoeugenol	1436	1454	164	0,05	0,06	0,01	0,04	0,04	0,31	0,01	0,01	0,37	0,53	0,00	0,28	0,02	0,02	0,02	0,09
013	Vanillyl methyl ketone	1495	1485	180	0,09	0,02	0,01	0,01	0,04	0,39	0,01	0,01	1,20	0	0,12	0,31	0,01	0,01	0,40	0,12
014	Mandelamide	1522	1536	107	0,07	0,46	0,00	0,04	0,02	1,05	0,00	0,01	0,05	0	0,05	0,13	0,01	0,01	0,11	0,17
015	3',5'-Dimethoxyacetophenone	1533		180	0,03	0,04	0,09	0,02	0,03	0,10	0,08	0,05	0,24	0	0,04	0,11	0,01	0,01	0,01	0,08
016	Butyrovanillone	1563	1592	151	0,24	0,19	0,07	0,12	0,20	1,50	0,08	0,14	1,21	1,94	0,66	0	0,01	0,01	0,01	0,29
017	Phenol, 2,6-dimethoxy-4-(2-propenyl)-	1561	1572	194	0,03	0,06	0,18	0,04	0,08	0,07	0,36	0,85	3,16	0	0,35	0,05	0,01	0,01	0,02	0,73
018	3,4,5-Trimethoxyphenol	1563		169	0,11	0,01	0,10	0,03	0,01	0,40	0,11	0,03	1,36	0	0,00	0,07	0,08	0,01	0,01	0,79
019	Benzenepropanol, 4-hydroxy-3-methoxy-	1620	1729	180	0,00	0,01	0,19	0,01	0,16	0,04	3,21	0,01	0,95	0,78	0,14	0,01	0,00	0,00	0,00	0,36
020	(E)-2,6-Dimethoxy-4-(prop-1-en-1-yl)phenol	1676	1704	194	0,10	0,07	0,71	0,06	0,10	0,28	0,56	0,05	0,23	2,18	0,04	0,08	0,01	0,03	0,03	0,31
021	2-Propanone, 1-hydroxy-3-(4-hydroxy-3-methoxyphenyl)-	1693	1736	196	0,01	0,02	0,12	0,01	0,01	0,03	0,14	0,01	0,33	0	0,01	0,01	0,01	0,01	0,01	1,31
022	Desaspidinol	1736		167	0,22	0,06	0,10	0,07	0,09	1,64	0,16	0,07	0,48	0	0,05	0,03	0,01	0,01	0,03	1,28
023	Tetradecanoic acid	1744	1752	228	0,00	0,10	0,39	0,06	0,11	0,00	0,37	0,01	0,12	0	0,04	0,02	0,01	0,01	0,01	1,29
024	3,5-Dimethoxy-4-hydroxyphenylacetic acid	1755	1744	167 212	0,19	0,02	0,00	0,02	0,04	0,50	0,00	0,03	0,00	0	0,04	0,01	0,01	0,01	0,04	0,00
025	Dehydrovomifoliol	1767	1800	124	0,04	0,04	0,07	0,01	0,05	0,06	0,02	0,04	0,07	0	0,00	0,01	0,01	0,01	0,06	0,97
026	Acetosyringon	1783	1744	181	0,52	0,06	0,32	0,82	0,10	1,22	0,03	0,01	1,95	0	0,03	0,09	0,01	0,01	0,13	0,49
027	3,4-Dimethoxycinnamic acid	1792	1926	208	0,04	0,17	0,43	0,09	0,10	0,03	0,41	0,73	0,88	0	0,25	0,01	0,04	0,01	0,05	0,05
028	Neophytadiene	1836	1842	123	0,07	0,81	0,25	0,12	0,83	0,03	0,66	0,05	0,24	2,72	0,04	0,01	0,01	0,01	0,01	0,17
029	Pentadecanoic acid	1838	1848	242	0,00	0,02	0,06	0,23	0,02	0,01	0,10	0,19	0,04	0,28	0,19	0,03	0,01	0,01	0,01	0,08
030	Neophytadiene 2	1839	1842	123	0,01	0,21	0,18	0,02	0,24	0,02	0,10	0,01	0,56	0,72	0,02	0,01	0,01	0,01	0,01	0,06
031	1-Hexadecanol	1869	1880	83	0,00	0,17	0,11	0,06	0,14	0,00	0,08	0,02	0,18	0,34	0,02	0,01	0,00	0,00	0,00	0,03
032	Neophytadiene 3	1883	1842	123	0,03	0,23	0,05	0,02	0,23	0,03	0,11	0,01	0,08	1,55	0,07	0,06	0,01	0,01	0,01	0,05
033	Hexadecanoic acid, methyl ester	1924	1953	236	0,00	0,02	0,04	0,06	0,02	0,01	0,62	0,01	0,19	0,72	0,00	0,01	0,01	0,00	0,00	0,08
034	Palmitoleic acid	1926	1926	74	0,00	0,15	0,51	0,05	0,18	0,03	0,27	0,30	0,56	0,46	0,03	0,01	0,01	0,01	0,01	0,19
035	Hexadecanoic acid	1943	1942	256	3,09	0,66	2,22	0,61	0,70	0,20	2,29	0,53	2,13	3,24	1,32	0,20	0,69	0,76	0,79	0,61
036	Heptadecanoic acid	2060	2071	37,270	0,00	0,03	0,09	0,01	0,03	0,01	0,13	0,01	0,01	0,41	0,00	0,01	0,01	0,01	0,01	0,04
037	Aucuparin	2050	2063	82	0,39	0,05	0,00	0,06	0,06	0,03	0,00	0,04	0,00	0	0,02	0,07	0,01	0,02	0,71	0,00
038	1-Octadecanol	2073	2074	97	0,15	0,03	0,08	0,07	0,04	0,07	0,06	0,05	0,01	0,29	0,05	0,05	0,01	0,01	0,01	0,02
039	Heneicosane	2098	2100	57	0,00	0,02	0,00	0,03	0,06	0,00	0,36	0,03	0,00	0	0,07	0	0,01	0,02	0,19	0,03
040	Phytol	2107	2114	71	5,94	0,52	0,10	0,09	0,56	2,70	0,10	0,09	3,24	16,36	0,11	0,95	0,60	0,12	0,13	0,89
041	Linoleic acid	2113	2112	280	3,47	0,06	0,37	0,05	0,13	0,43	0,53	0,04	0,83	4,40	1,38	0,08	0,01	0,01	0,01	0,21
042	Linolenic acid	2117	2115	79	1,86	0,27	0,36	0,12	0,41	0,33	1,31	0,27	0,41	1,57	0,60	0,05	0,03	0,01	0,01	0,05
043	Oleic acid	2119	2134	264	2,64	0,13	0,43	0,13	0,17	0,11	0,22	0,17	0,62	0	0,51	0,25	0,02	0,02	0,20	0,16
044	Octadecanoic acid	2143	2153	284	1,63	0,08	0,91	0,12	0,14	0,27	0,53	0,05	1,53	1,24	0,22	0,01	0,01	0,01	0,01	0,23
045	2'-Methoxyaucuparin	2169		260	0,07	0,00	0,00	0,00	0,01	0,03	0,00	0,01	0,01	0	0,00	0,01	0,00	0,01	0,01	0,01
046	Docosane	2201	2200	57	0,00	0,01	0,09	0,02	0,01	0,00	0,02	0,01	0,03	0	0,01	0,01	0,01	0,01	0,00	0,03
047	Benzyl β-D-glucoside	2228		91	0,21	0,03	0,11	0,13	0,04	2,54	0,10	0,13	0,03	2,81	0,16	0,09	0,01	0,00	0,00	24,28
048	1-Eicosanol	2275	2273	97	0,07	0,03	0,13	0,04	0,04	0,16	0,04	0,05	0,24	0,23	0,04	0,03	0,01	0,01	0,01	0,08
049	Tricosane	2304	2304	57	0,02	0,04	0,03	0,05	0,05	0,00	0,03	0,06	0,03	0,26	0,05	0,02	0,02	0,03	0,03	0,09

050	4,8,12,16-Tetramethylheptadecan-4-olide	2331	2364	99	0,03	0,01	0,05	0,01	0,01	0,25	0,01	0,01	0,11	4,31	0,01	0,01	0,01	0,01	0,01	0,02
051	Eicosanoic acid	2343	2366	312	0,03	0,04	0,18	0,11	0,05	0,15	0,10	0,07	0,52	0,45	0,06	0,04	0,01	0,02	0,02	0,09
052	1-Heneicosanol	2373	0	97	0,03	0,01	0,00	0,03	0,02	0,00	0,00	0,01	0,00		0,00	0,02	0,01	0,01	0,00	0,00
053	Tetracosane	2404	2404	57	0,00	0,00	0,11	0,00	0,00	0,00	0,11	0	0,03	0,33	0,00	0	0,01	0,02	0,02	0,00
054	1,1'-Biphenyl, 6-hydroxy-4,2',3',4'-tetramethoxy-	2413	0	290	0,09	0,07	0,00	0,01	0,00	0,00	0,00	0,39	0,00	0	0,00	0,01	0,01	0,00	0,00	0,00
055	Octadecanamide, N-ethyl	2434		87	0,12	0,02	0,00	0,00	0,03	0,00	0,00	0	0,00	0,56	0,02	0	0,01	0,01	0,01	0,00
056	Behenic alcohol	2472	2457	385	2,21	0,78	0,18	1,14	0,88	0,87	1,13	0,73	0,03	0,57	0,69	0,77	0,14	0,17	0,18	0,64
057	Dehydroabietic acid	2477	2475	97	0,21	0,58	1,97	0,13	0,58	0,00	0,30	0,18	0,50		0,00	0	0,02	0,35	0,00	0,26
058	Prunasin	2477	0	117	16,09	41,32	7,63	36,68	30,92	3,10	1,61	36,62	2,14	3,48	6,80	7,16	0,21	2,09	2,17	1,05
059	Pentacosane	2501	2504	57	0,00	0,09	0,18	0,26	0,09	0,00	0,24	0,01	0,01	1,03	0,00	0,41	0,03	0,01	0,01	0,01
060	Docosanoic acid	2546	2567	340	0,16	0,18	0,13	0,40	0,34	1,77	0,22	0,19	1,00	0,10	0,23	0,18	0,03	0,10	0,11	0,17
061	Mm 318 $C_{20}H_{30}O_3$	2604	2366	207	0,18	0,06	0,04	0,05	0,05	0,26	0,00	0,05	0,04	0	0,11	0,21	0,23	0,36	0,00	0,15
062	Hexacosane	2600	2600	57	0,00	0,00	0,32	0,00	0,00	0,00	0,44	0	0,00	0,33	0,00	0	0,01	0,01	0,01	0,09
063	Tetracosanal	2614	2614	334	0,00	0,04	0,00	0,01	0,03	0,00	0,00	0,01	0,00		0,00	0,01	0,00	0,01	0,01	0,00
064	1-Tetracosanol	2679	2678	97	0,08	1,73	0,03	2,83	1,86	0,15	0,10	1,76	0,87	3,73	1,92	0,46	0,24	0,27	0,28	1,34
065	Heptacosane	2698	2704	57	0,01	0,11	0,35	0,01	0,15	0,00	0,62	0,01	0,01	1,14	0,04	0,01	0,05	0,04	0,04	0,01
066	Tetracosanoic acid	2744	2685	368	0,02	0,09	0,00	0,22	0,10	0,02	0,00	0,01	0,00		0,00	0,01	0,01	0,02	0,14	0,03
067	Mm 334 c 95, 107 $C_{20}H_{30}O_4$	2787	-	207	0,22	0,05	0,01	0,00	0,04	0,54	0,00	0,02	0,00	0,26	0,11	0,10	0,09	0,07	0,07	0,16
068	Mm 332 c 97 $C_{20}H_{28}O_4$	2801	-	207	0,80	0,07	0,04	0,09	0,06	0,43	0,00	0,09	0,00	0,23	2,86	0,33	0,38	0,59	0,62	0,25
069	Tetracosyl acetate	2804	2808	97	0,09	0,11	0,11	0,04	0,07	0,04	0,15	0,09	0,32		0,26	0,09	0,01	0,00	0,06	0,00
070	Mm 334 c 95, 107 $C_{20}H_{30}O_4$	2804	0	207	0,77	0,97	0,15	2,28	3,21	2,14	0,00	1,06	0,04	0	0,61	2,99	3,06	4,48	4,05	0,78
071	Squalene	2811	2817	69	2,32	2,07	8,04	1,73	5,76	1,55	7,93	2,13	7,78	1,56	2,65	1,05	0,45	0,94	1,03	4,56
072	Hexacosanal		2832	57, 82	0,00	0,60	0	0	2,13	0	0	0	0	0	0	0	0	1,85	2,21	0,21
073	Tocospiro A	2825	2860	419	0,03	0,00	0,01	0,01	0,01	0,29	0,03	0,01	0,02	0	0,01	0,01	0,06	0,00	0,76	0,01
074	Mm 332 $C_{20}H_{28}O_4$	2831	-	207	0,41	0,07	0,01	0,10	0,08	0,58	0,00	0,11	0,01	0	0,74	0,28	0,31	0,50	0,66	0,42
075	Tocospiro B	2845	2882	419	0,03	0,01	0,01	0,01	0,01	0,17	0,02	0	0,03	0	0,00	0,01	0,06	0,00	0,93	0,01
076	1-Hexacosanol	2883	2852	97	6,32	4,97	1,30	2,87	5,65	2,35	1,42	2,17	0,04	3,10	4,78	0,29	2,74	7,81	8,13	0,02
077	Nonacosane	2900	2900	57	0,00	0,16	0,12	0,09	0,17	0,00	1,18	0,06	0,05	1,73	0,09	0,11	0,07	0,05	0,05	0,02
078	A-Neooleana-3(5),12-diene	2904		365	0,35	0,07	0,65	0,12	0,30	0,17	0,54	0,39	0,64	3,72	0,62	0,08	0,51	0,68	0,44	0,10
079	Diterpenoic acid Mm Mm358	2914		207	0,32	0,12	0,00	0,09	0,08	4,18	0,00	0,10	0,00	0	0,45	0,54	0,31	0,54	0,02	0,14
080	Stigmastan-3,5,22-trien	3025	2981	394	0,03	0,15	0,00	0,05	0,15	0,02	0,02	0,06	0,84	0,12	0,07	0	0,01	0,01	0,01	0,15
081	Stigmasta-3,5-diene	3046		396	0,07	0,06	0,09	0,14	0,06	0,10	0,14	0,10	0,59	0	0,09	0,09	0,41	0,18	0,02	0,14
082	Cholesterol	3054	3087	386	0,07	0,03	0,10	0,03	0,04	0,20	0,11	0,04	0,49	0,18	0,04	0,05	0,13	0,01	0,01	0,03
083	Vitamin E	3084	3138	430	1,23	3,84	2,96	1,13	4,18	0,76	2,73	0,85	0,97	0	0,13	1,06	1,26	0,03	0,13	1,16
084	Campesterol	3150	3131	400	0,03	0,02	0,87	0,04	0,02	0,03	0,46	0,04	0,00	0,54	0,05	0,04	0,17	0,00	0,15	0,00
085	β-Sitosterol	3232	3230	414	2,13	3,55	24,57	3,53	3,56	1,90	22,79	2,98	26,62	12,37	3,41	3,44	5,69	0,94	0,70	2,19
086	Fucosterol	3264	3293	314	2,91	0,45	2,10	0,00	0,00	0,00	4,85	1,42	4,23	0	0,01	1,05	1,54	0,00	0,00	1,24
087	β-Amyrin	3245	3337	218	0,23	1,39	1,10	0,47	0,45	0,00	0,00	0,49	0,00	0,98	0,38	1,60	1,03	0,28	0,43	0,62
088	Lup-20(29)-en-3-one	3267	3384	424	2,55	0,28	0,33	0,35	0,35	0,80	0,90	0,42	2,36	0	0,54	0	0,00	0,91	0,85	0,00
089	α-Amyrin	3275	3376	218	0,00	1,06	0,05	1,15	0,00	0,09	0,00	0	0,00	0,71	0,00	0	0,00	0,00	0,00	0,71
090	Lupeol	3288	3270	426	13,00	20,93	0,75	32,57	20,73	29,27	2,54	35,70	5,22	0	34,65	67,73	70,06	67,14	61,43	32,46
091	Not identificated Mm 444	3292		125,175	0,88	1,28	0,00	0,54	0,96	1,43	0,00	1,19	0,00	0	0,97	0	0,00	0,99	1,53	0,00
092	Stigmasta-3,5-dien-7-one	3305		174	0,17	0,11	0,73	0,20	0,09	0,00	0,20	0,08	0,00	0	0,00	0,82	0,11	0,71	0,00	0,01
093	Not identificated Mm 442	3314		125	0,77	0,93	0,00	0,24	0,88	1,37	0,00	0,60	0,00	0	0,00	0	0,81	1,03	1,46	1,64
094	Allobetulin	3320		189	1,82	0,54	0,00	0,34	0,51	1,76	0,00	0,42	0,00	0	0,07	0	0,39	0,20	1,88	1,71
095	β-Sitostenone	3339	3483	412	0,07	0,19	0,15	0,15	0,10	0,45	0,38	0,07	0,00	0	0,25	0,16	0,11	0,39	0,47	0,38

096	Lupeol acetate	3409		189	0,00	0,11	0,17	1,02	0,34	0,47	0	1,11	0,00	0	0,68	0	0,59	0,19	0,12	2,47
097	Taraxasterol	3437		189	1,45	1,13	0	0,76	1,56	3,02	0	0,65	0,00	0	1,60	0	0,27	1,56	0,42	0,13
098	Dotriacontanal	3442	3451*	82	1,87	0,18	0	0,81	0,00	1,00	0	0	0,00	0	0,73	0	0,26	0,23	0,26	0,00
099	Not identificated	3448		428	1,55	1,00	0	0,09	1,42	0	0	0,97	0,00	0	1,54	0,43	0,32	0,08	0,00	0,00
100	Betulinaldehyde	3551		189	0,21	0,29	0	0,66	0,00	0	0	0,30	0,00	0	0,70	0,39	0,90	0,12	0,00	0,00
101	Betulin	3572		189	0,04	0,06	0	0,18	2,94	0	0	0,07	2,07	0	7,90	0,31	1,50	1,40	0,00	0,00

*estimated by RI_{lit} of homologues

Table S3. Compounds identified in extracts from *S.aucuparia* bark by GC/MS (winter gathering samples)

#	compounds	RI	RI_{lit}	Q_m	1w	2w	3w	4w	5w	6w	7w	8w	9w	10w	11w	12w	13w	14w	15w	16w
001	Benzaldehyde	957	965	106	6,87	17,75	0,61	7,68	2,34	34,13	38,68	5,51	0,06	0,62	0,12	0,54	9,30	5,01	0,71	0,11
002	Benzyl alcohol	1019	1026	108	0,09	0,55	0,01	0,05	0,06	0	0,20	0,24	0	0,21	0	0	0,11	0	0,17	0
003	Benzeneacetonitrile, α-oxo-	1077		131	0,15	0,78	0,01	0,19	0,17	1,56	0,14	0	0	0,01	0	0,07	0,14	0,13	0,01	0
004	Benzeneacetic acid, α-methoxy-, methyl ester	1088	-	121	1,70	0,57	0,01	0,04	0,07	0,58	0,24	4,55	0	0,10	0	0,01	1,12	0,19	0,07	0,01
005	Benzeneacetonitrile	1111	1103	117	0,09	0,38	0,03	0,26	1,16	1,18	0,67	0,49	0	0,02	0	0,07	0,08	0,26	0,02	0
006	Benzoic acid	1164	1159	122	0,62	1,11	1,52	0,68	7	1,81	1,13	0,90	0	0,55	0	0,05	0,28	1,80	0,54	0
007	Coumaran	1192	1188	120	0,07	0,05	0,26	0,08	0,05	0	0,07	0,01	0	0,66	0	0,01	0,01	0,06	0,25	0
008	4-Vinyl-2-methoxyphenol	1298	1317	135	0,15	0,05	1,05	0,06	0,02	0,01	0,07	0,08	0	0,21	0	0,04	0,01	0,06	0,17	0
009	Phenol, 2,6-dimethoxy-	1320	1320	154	0,01	0,01	0,02	0,01	0,01	0,04	0,07	0,01	0	0,04	0	0,01	0,01	0,05	0,04	0
010	Eugenol	1341	1335	164	0,04	0,05	0,47	0,08	0,23	0,09	0,13	0,22	0	0,02	0	0,31	0,15	0,32	0,06	0
011	Vanillin	1367	1357	152	0,10	0,05	0,01	0,04	0,03	0,16	0,06	0,66	0	0,01	0	0,01	0,03	0,02	0,02	0
012	trans-Isoeugenol	1436	1454	164	0,10	0,06	0,01	0,07	0,06	0,19	0,10	0,15	0	0,02	0	0,02	0,08	0,14	0,03	0
013	Vanillyl methyl ketone	1495	1485	180	0,07	0,08	0,01	0,12	0,14	0,12	0,16	0,09	0,01	0,01	0,01	0,13	0,01	0,21	0,01	0
014	Mandelamide	1522	1536	107	0,07	0,08	0,07	0,12	0,16	0	0,34	0,08	0	0,01	0	0,02	0,19	0,19	0,04	0
015	3',5'-Dimethoxyacetophenone	1533		180	0,01	0,03	0,09	0,01	0,03	0,04	0,14	0,01	0,01	0,15	0	0,01	0,01	0,07	0,10	0
016	Butyrovanillone	1563	1592	151	0,03	0,26	0,15	0,48	0,79	1,57	1,11	0,14	0	0,21	0	0,15	0,19	1,13	0,89	0
017	Phenol, 2,6-dimethoxy-4-(2-propenyl)-	1561	1572	194	0,04	0,02	0,29	0,06	0,02	0	0,01	0,02	0	0,10	0	0,09	0,03	0,37	0,16	0
018	3,4,5-Trimethoxyphenol	1563		169	0	0	0,01	0,01	0	0,01	0,06	0	0	0,01	0	0,03	0,16	0,02	0,01	0
019	Benzenepropanol, 4-hydroxy-3-methoxy-	1620	1729	180	0	0,06	0,01	0,01	0,03	0	0,06	0,01	0	0,01	0	0,14	0,01	0,11	0,01	0
020	(E)-2,6-Dimethoxy-4-(prop-1-en-1-yl)phenol	1676	1704	194	0,03	0,02	0,01	0,02	0,03	0,07	0,07	0,04	0,01	0,01	0	0,02	0,03	0,10	0,02	0
021	2-Propanone, 1-hydroxy-3-(4-hydroxy-3-methoxyphenyl)-	1693	1736	196	0,01	0,01	0,15	0,01	0,01	0	0	0,01	0	0,35	0	0,01	0	0,02	0,41	0
022	Desaspidinol	1736		167	0,02	0,05	0,01	0,04	0,10	0,08	0,01	0,03	0	0,01	0	0,08	0,03	0,38	0,01	0
023	Tetradecanoic acid	1744	1752	228	0,01	0,01	0,17	0,01	0,01	0	0,33	0,01	0,02	0,24	0,01	0,01	0,01	0,03	0,22	0
024	3,5-Dimethoxy-4-hydroxyphenylacetic acid	1755	1744	167 212	0,02	0,05	0,09	0,02	0,06	0	0,03	0,04	0	0,05	0	0,03	0,08	0,32	0,12	0
025	Dehydrovomifoliol	1767	1800	124	0,02	0,03	0,21	0,02	0,05	0,25	0,27	0,07	0	0,42	0	0,05	0,19	0,24	0,39	0
026	Acetosyringon	1783	1744	181	0,08	0,08	0	0,06	0,13	0,58	0,10	0,12	0	0,01	0	0,09	0,03	0,63	0,06	0
027	3,4-Dimethoxycinnamic acid	1792	1926	208	0,01	0,01	0,01	0,05	0,05	0,49	0,47	0,04	0	0,01	0	0,02	0,02	0,24	0,01	0
028	Neophytadiene	1836	1842	123	0,04	0	0,01	0,04	0,14	0,37	0,26	0,09	0,32	0,01	0,08	0,01	0,15	0,11	0,01	0,39
029	Pentadecanoic acid	1838	1848	242	0,01	0,01	0,01	0,01	0,01	0	0,17	0,01	0	0,01	0	0,01	0,01	0,02	0,02	0
030	Neophytadiene 2	1839	1842	123	0,03	0	0,01	0,01	0,02	0,17	0,01	0,03	0,07	0,01	0,02	0,01	0,04	0,08	0,01	0,06
031	1-Hexadecanol	1869	1880	83	0,03	0,03	0,01	0,06	0,05	0,25	0	0	0,28	0,01	0	0,01	0,01	0,02	0,01	0

032	Neophytadiene 3	1883	1842	123	0,01	0	0,01	0	0,03	0,11	0	0,05	0,14	0	0,03	0	0,04	0,03	0	0,09
033	Hexadecanoic acid, methyl ester	1924	1953	236	0,13	0,07	0,03	0,01	0,06	0	0,04	0,34	0,85	0,03	0,01	0,02	0,03	0,08	0,01	0,05
034	Palmitoleic acid	1926	1926	74	0,01	0,03	0,01	0,31	0,07	0	0,16	0,02	0	0,01	0	0,01	0,03	0,11	0,01	0
035	n-Hexadecanoic acid	1943	1942	256	0,19	2,19	0,06	0,29	1,80	0,30	0,82	0,35	0,18	0,04	0,11	0,26	0,55	1,72	0,07	0,11
036	Heptadecanoic acid	2060	2071	37,270	0	0,01	0	0,01	0	0	0,01	0,01	0	0	0	0,01	0,01	0,01	0,01	0
037	Aucuparin	2050	2063	82	0,30	0,19	0,03	0,16	0,39	0	0,09	0,78	0	0,04	0	0,04	0,13	0,51	0,04	0
038	1-Octadecanol	2073	2074	97	0,04	0,07	0,03	0,04	0,28	0	0,06	0	0,40	0,01	0,48	0,01	0,07	0,17	0,09	0,10
039	Heneicosane	2098	2100	57	0	0	0	0	0	0	0,11	0,15	0	0	0	0,01	0	0	0	0,04
040	Phytol	2107	2114	71	1,97	1	0,62	1,31	2,12	4,57	0,09	4,12	14,24	0,38	1,07	0,60	1,05	1,21	0,40	4,07
041	Linoleic acid	2113	2112	280	0,02	0,03	0,02	0,01	0,03	0,03	0,06	0,04	0	0,01	0,01	0,01	0,02	0,05	0,02	0
042	Linolenic acid	2117	2115	79	0,20	0,27	0,01	0,07	0,51	0,50	0,41	0,65	0	0,01	0,02	0,03	0,15	0,29	0,01	0
043	Oleic acid	2119	2134	264	0,01	0,01	0,01	0,01	0,01	0,01	0,03	0,01	0	0,01	0,01	0,01	0,01	0,02	0,01	0
044	Octadecanoic acid	2143	2153	284	0,03	0,13	0,01	0,06	0,16	0,07	0,20	0,15	0,02	0,01	0,01	0,02	0,08	0,22	0,02	0
045	2'-Methoxyaucuparin	2169		260	0,01	0,01	0,07	0,01	0	0	0,06	0	0	0,08	0	0,02	0,27	0,14	0,25	0
046	Docosane	2201	2200	57	0,01	0,02	0	0	0,03	0	0	0	0	0	0	0	0,01	0,02	0,01	0,06
047	Benzyl β-D-glucoside	2228		91	0,19	0,41	0,27	0,51	0,21	0,21	0,18	1,20	0	0,57	0	0,04	0,10	2,48	0,40	0
048	1-Eicosanol	2275	2273	97	0	0	0,01	0	0	0	0	0	0,46	0,01	0,12	0	0	0	0,03	0
049	Tricosane	2304	2304	57	0,05	0,03	0	0	0,03	0,34	0,10	0	0,16	0	0,05	0	0,02	0,01	0,03	0,15
050	4,8,12,16-Tetramethylheptadecan-4-olide	2331	2364	99	0,04	0,10	0,02	0,01	0,08	0,04	0,01	0,03	0,83	0,03	0,30	0,01	0,07	0,18	0,05	0,15
051	Eicosanoic acid	2343	2366	312	0,02	0,13	0,01	0,05	0,09	0	0,20	0,33	0	0,01	0	0,01	0,08	0,14	0,02	0
052	1-Heneicosanol	2373	0	97	0	0,07	0,01	0	0	0	0	0	0	0,01	0	0	0,01	0,01	0,01	0
053	Tetracosane	2404	2404	57	0,01	0	0	0	0	0	0	0	0,09	0	0,02	0	0	0	0,01	0,04
054	1,1'-Biphenyl, 6-hydroxy-4,2',3',4'-tetramethoxy-	2413	0	290	0,01	0,01	0,01	0,01	0	0	0,10	0,01	0	0,01	0	0,02	0,16	0,05	0,04	0
055	Octadecanamide, N-ethyl	2434		87	0	0,03	0	0	0	0	0	0	0	0	0,01	0	0,03	0,01	0,01	0
056	Behenic alcohol	2472	2457	385	0	0	0,05	0	0	0	0	1,11	0,18	0,04	0,14	0	0	0	0,06	5,65
057	Dehydroabietic acid	2477	2475	97	0	0	0	0	0	0	0,03	0	0,02	0	0	0	0	0	0	0
058	Prunasin	2477	0	117	44,46	62,50	22,03	54,99	73,13	44,23	36,18	19,75	0,05	11,86	0	20,27	30,01	31,79	3,42	0
059	Pentacosane	2501	2504	57	0	0	0	0	0	0	0	0	0,13	0,01	0,03	0	0	0	0,01	0
060	Docosanoic acid	2546	2567	340	0,10	0,45	0,03	0,20	0,29	0	0,70	0,50	0	0,03	0	0,03	0,22	0,42	0,09	0
061	Mm 318 $C_{20}H_{30}O_3$	2604	2366	207	0,33	0	0,19	0,10	0,28	0	0,01	0,05	0,23	0,93	0,81	0,13	0,05	0,51	2,38	0,52
062	Hexacosane	2600	2600	57	0	0	0,01	0	0	0	0	0	0,11	0,01	0,04	0	0	0	0,03	0,11
063	Tetracosanal	2614	2614	334	0,01	0,03	0	0,01	0,02	0	0,04	0,01	0	0	0,01	0	0	0,01	0	0
064	1-Tetracosanol	2679	2678	97	2,09	0,29	0,27	0	0,19	0	0	1,35	0	0,31	0	0	0,35	0,46	0,38	0,27
065	Heptacosane	2698	2704	57	0	0	0,01	0	0	0	0	0	0,03	0,02	0,01	0	0	0	0,01	0,06
066	Tetracosanoic acid	2744	2685	368	0	0,08	0,05	0,04	0,09	0	0,17	0,08	0	0,08	0	0	0,03	0,06	0,16	0
067	Mm 334 c 95, 107 $C_{20}H_{30}O_4$	2787	-	207	0,54	0,30	0,69	0,25	0,16	0	0	0,09	0,07	2,93	1,21	0,16	0,06	0,53	5,69	0,18
068	Mm 332 c 97 $C_{20}H_{28}O_4$	2801	-	207	0,72	0	0,51	0,42	0,34	0,56	0	0,17	0,31	1,53	1,53	3,10	0,07	0,65	1,69	0,56
069	Tetracosyl acetate	2804	2808	97	0	0,02	0	0	0,03	0	0	0,35	0	0	1,44	0	0,01	0,03	0	0
070	Mm 334 c 95, 107 $C_{20}H_{30}O_4$	2804	0	207	3,26	0,22	3,36	1,48	0,17	0	0	1,13	0	4,40	20,38	2,34	0,84	3,87	12,42	2,49
071	Squalene	2811	2817	69	0,84	1,35	6,97	1,17	2,72	1,39	8,45	0,63	0,67	4,92	0	1,02	0,86	4,66	9,50	14,19
072	Hexacosanal		2832	57, 82	0	0	0	0	0	0	0	0	0	0	0	0	0	0	0	0
073	Tocospiro A	2825	2860	419	0,09	0,19	0,23	0	0,10	0	0	0,48	0,14	0,28	0	0,02	0,15	0,24	0,51	0
074	Mm 332 $C_{20}H_{28}O_4$	2831	-	207	0,72	0	0,47	0,44	0,12	0,69	0	0,23	0	1,48	4,37	0,16	0,08	0,85	1,25	0,84
075	Tocospiro B	2845	2882	419	0,22	0,40	0,17	0	0,21	0	0	0,76	0,13	0,25	0	0,03	0,19	0,45	0,51	1,38
076	1-Hexacosanol	2883	2852	97	0,73	0,08	0	0,06	0,01	0	0	0,36	0	0	0	0,17	0,13	0,08	0	0

077	Nonacosane	2900	2900	57	0,02	0	0	0	0	0	0	0	0,07	0	0,03	0	0,01	0	0	0
078	A-Neooleana-3(5),12-diene	2904		365	0,04	0	0,03	0	0	0	0	0,18	0	0	0	0,03	0,08	0,01	0,01	0
079	Diterpenoic acid Mm 358	2914		207	2,59	0,38	2,68	1,73	0,37	1,07	0	0,58	0,27	14,08	28,19	0,57	0,12	2,78	22,43	0,73
080	Stigmastan-3,5,22-trien	3025	2981	394	0,08	0,12	0,33	0,07	0,14	0,30	0,13	0,23	0	0,38	0,27	0,06	0,15	0,33	0,89	0,53
081	Stigmasta-3,5-diene	3046		396	0,02	0,06	0,07	0,05	0,07	0	0	0,12	0	0	0,15	0,03	0,04	0,07	0	0,15
082	Cholesterol	3054	3087	386	0	0	0	0	0	0	0	0,01	0	0	0	0,03	0	0	0,02	0
083	Vitamin E	3084	3138	430	0,27	0,68	4,80	1,48	1,21	1,51	4,58	0,27	2,43	2,82	0	0,43	0,33	0,57	7,91	2,81
084	Campesterol	3150	3131	400	0,04	0	0	0	0	0	0	0,11	4,59	0,05	0	0	0	0	0,07	0
085	β-Sitosterol	3232	3230	414	1,16	0	0,69	1,73	0,01	0	0	11,12	0	0,44	0,92	1,85	1,51	0,97	1,03	0
086	Fucosterol	3264	3293	314	0,21	0	0,03	0	0	0	0	0,45	0	0,02	0	0	0,46	0	0,03	1,27
087	β-Amyrin	3245	3337	218	0,19	0	0,05	1,58	0	0	0	2,03	46,97	0,13	0	0,71	1,66	0,29	0,03	0
088	Lup-20(29)-en-3-one	3267	3384	424	0	0,44	0,08	0,43	0,24	0	0,94	0,42	0	0,08	1,69	1,22	0,23	0,94	0,10	0
089	α-Amyrin	3275	3376	218	0	0	0,05	0	0	0	0	0	0	0,09	0	0	0	0	0,06	0
090	Lupeol	3288	3270	426	22,33	4,80	48,82	13,42	0,71	0	0	34,16	25,47	45,59	28,21	55,51	46,81	38,38	20,21	59,23
091	Not identificated Mm 444	3292		125,175	0,24	0	0,51	0,28	0	0	0	1,66	0,91	2,13	0,66	1,16	0,87	0,62	0,82	0
092	Stigmasta-3,5-dien-7-one	3305		174	0,25	0	0,09	0,20	0	0	0,21	0,08	0	0,08	0	0	0,12	0,74	0,13	0
093	Not identificated Mm 442	3314		125	0,16	0	0	0,31	0	0	0	0,34	0,44	0,18	0,23	0,46	0,32	0	0	0
094	Allobetulin	3320		189	0	0	0,23	0	0	0	0	0,35	0	0,16	0,34	0	0,25	0,55	0	0
095	β-Sitostenone	3339	3483	412	0,23	0,21	0,05	0,20	0,15	0	0,40	0,07	0	0,04	0	0,16	0,12	0,40	0,04	0,52
096	Lupeol acetate	3409		189	0	0	0	1,52	0	0	0	0,50	0	0,59	0	1,75	0	0	0	0
097	Taraxasterol	3437		189	1,33	0	0	0	0	0	0	0,41	0	0	0	0	0	1,61	0	0
098	Dotriacontanal	3442	3451*	82	0,19	0	0,19	0,25	0	0	0	0,42	0	0,21	0,34	1,28	0,41	0,51	2,36	0
099	Not identificated	3448		428	0,24	0	0,34	0	0	0	0	0,17	0	0	0	0,43	0,10	0	2,70	0,40
100	Betulinaldehyde	3551		189	1,69	0	0	3,35	0	0	0	0	0	0	0	1,38	0,31	2,19	0	0,52
101	Betulin	3572		189	0,08	0	0	0	0	0	0	0,09	0	0	0	2,98	0	0	0	0

**estimated by RI_{lit} of homologues
